# Anticrossing of A Plasmonic Nanoresonator Mode and A Single Quantum Dot at Room Temperature

**DOI:** 10.1002/advs.202506676

**Published:** 2025-08-25

**Authors:** Daniel Friedrich, Jin Qin, Benedikt Schurr, Tommaso Tufarelli, Heiko Groß, Bert Hecht

**Affiliations:** ^1^ Nano‐Optics and Biophotonics Group Experimentelle Physik 5 Physikalisches Institut Universität Würzburg D‐97074 Würzburg Germany; ^2^ Independent Researcher Beeston NG9 UK

**Keywords:** anticrossing, plasmonic cavity, single quantum emitter, strong coupling

## Abstract

Strong coupling between a single quantum emitter and a resonant plasmonic mode at room temperature is vital for quantum information processing and sensing. Beating dephasing in these systems by ultrafast energy transfer requires coupling single emitters to a plasmonic nanoresonator with ultrasmall mode volume and optimal spectral overlap. Typically, strong coupling is inferred from normal mode splittings in luminescence spectra, offering rough estimates of coupling strength. However, achieving a full anticrossing and characterizing uncoupled components in advance is challenging. Here, the oxygen‐dependent blue‐shift of CdSe/ZnS quantum dots, recorded at 33 ms time resolution, is leveraged to deterministically tune their transition energy across a scanning plasmonic slit resonator to yield a complete anticrossing, after characterizing both uncoupled states beforehand. The findings provide clear evidence of strong coupling at room temperature, with a Rabi splitting at zero detuning of 100meV, consistent with theoretical modeling. This work advances the development of deterministic plexitonic devices utilizing single‐photon nonlinearities at ambient conditions.

## Graphical abstract

Using the oxygen‐dependent blue‐shift in CdSe/ZnS quantum dots, their emission is deterministically tuned across the resonance of a plasmonic slit resonator, achieving a complete anticrossing with a 100 meV Rabi splitting. By pre‐characterizing the uncoupled states, strong coupling at room temperature is confirmed. These results agree with theoretical models and support deterministic plexitonic devices with single‐photon nonlinearities.

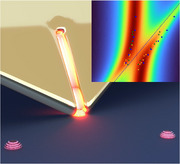

## 1 Introduction

A single two‐level system strongly coupled to a single mode of an electromagnetic field of a resonator exhibits a characteristic single‐photon nonlinearity in its energy spectrum. The observed splitting of the emerging eigenmodes of the strongly‐coupled system scales with the square root of the number of photons in the system. That is, the addition of a single photon to the system changes its response to a follow‐up photon. Such behavior, if realized at ambient conditions, holds promise to overcome the need for cryogenics in quantum information processing and quantum sensing thus unlocking an enormous potential for applications.^[^ ^1^, ^2^, ^3^, ^4^ ^]^

To achieve strong coupling at ambient conditions, many studies exploited the square‐root scaling of the coupling strength with the number of emitters that couple to the same cavity mode, e.g., by making use of J‐aggregates or otherwise densely‐packed emitter systems.^[^ ^5^, ^6^, ^7^, ^8^, ^9^ ^]^ In such systems a coupled bright state of many emitters hybridizes with the cavity mode leading to interesting collective effects, such as enhanced photo‐chemistry, enhanced conductivity and possibly light‐induced superconductivity.^[^ ^10^ ^]^ However, the price to pay in these systems is that the single‐photon nonlinearity of the associated Tavis‐Cummings model decreases with the number of emitters. To retain the single‐photon nonlinearity, there has been a quest for solid evidence of reaching single‐emitter strong light‐matter coupling (SC) at ambient conditions.^[^ ^11^, ^12^ ^]^ Experiments are based on the idea that the ultrasmall mode volumes of plasmonic nanoresonators should lead to strong coupling in spite of intrinsic losses and dephasing leading to low quality factors.

The necessary small mode volume of the nanoresonator at ambient conditions requires solutions for positioning a single emitter inside the hotspot of a plasmonic nanoresonator with nanometer precision. One approach has been to use a random distribution of single emitters that are spread at low concentration on top of plasmonic nanoresonators, such as arrays of bow‐tie antenna,^[^ ^4^, ^13^, ^14^ ^]^ nanoparticle‐on‐mirror geometries,^[^ ^15^, ^16^ ^]^ or plasmonic nanodimers.^[^ ^17^ ^]^ Scanning‐probe techniques, which position a plasmonic nanoresonator with nanometer accuracy on a surface also have proven useful to achieve the necessary positioning accuracy while providing the possibility to vary the coupling strength deliberately at any time to study the uncoupled entities.^[^ ^18^ ^]^ In all experiments photoluminescence spectra (PL) that exhibit two clearly split peaks shifted red and blue with respect to the uncoupled resonance of cavity and emitter system are generally presented as proof of strong coupling. However, the observation of a single split spectrum for a fixed detuning, only, may be considered a weak proof for SC since vast room is left for alternative explanations.^[^ ^11^ ^]^ Observation of anticrossing of emitter and resonator resonance together with a characterization of the uncoupled partners represents a much stronger and well‐established evidence for SC.^[^ ^2^, ^19^ ^]^ Yet, in room‐temperature SC experiments so far, anticrossing data have either been missing, or have been obtained by stitching measurements from different coupled systems, which clearly falls behind the ideal single emitter ‐ single resonator experiment.^[^ ^15^, ^16^, ^20^, ^21^ ^]^ Besides proving strong coupling, anticrossing curves are also necessary to quantify the coupling strength at zero detuning because a possible nonzero detuning between the cavity and two‐level system will increase the observed splitting of the spectra, thus mimicking a larger coupling strength. Furthermore, in all single‐emitter strong coupling experiments reported so far, only a statistical characterization of the emitter and resonator has been performed. This leaves considerable uncertainties regarding the properties of individual systems and hampers the exclusion of detuning between the emitter and resonator, resulting in only qualitative estimates of the coupling strength.

**Figure 1:**
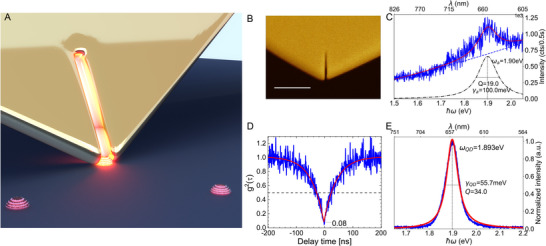
Experimental setup for strong coupling experiments and characterization of uncoupled plasmonic nanoresonators (PNR) and single Qdots. A) A gold microplatelet scanning PNR in close contact to a quantum dot (artistic representation). B) SEM image of a fabricated PNR. (Scale bar: 500 nm) C) Photoluminescence spectrum of a bare PNR excited with green laser (532 nm) excitation (power: 500 µW, intensity: 4.4× 10^9^ W m^−2^). The spectrum can be fitted a combination of with an Exponential (green dashed line, indicating the PL background signal) and a Lorentzian (black dot‐dashed line, indicating the PNR resonance and quality factor). The PL signal is recorded with an integration time of 0.5 s and an EMCCD gain of 150. D) Second‐order autocorrelation function (*g* ^2^(τ)) obtained by evaluating single photon arrival times to verify if the PL signal comes from a single Qdot or clusters. The dip at zero time delay (0.08) signifies the presence of antibunching characteristic for a single emitter. E) A single CdSe/ZnS Qdot emission spectrum excited by a green laser (532 nm, power: 1 µW, intensity: 8.8× 10^6^ W m^−2^).

Here, we demonstrate complete anticrossing of the PL peak of a single CdSe/ZnS semiconductor quantum dot (Qdot) and the second‐order resonance of a scanning plasmonic nanoslit resonator at room temperature, which have both been characterized separately before the coupling experiment (**Figure** **1**). Tuning of the Qdot PL is achieved using a light‐induced spectral shift in presence of oxygen that increases linearly with time. The obtained anticrossing allow us to extract the light‐matter coupling strength at zero detuning with excellent precision, capitalizing on an unprecedented time resolution of 33 ms, which suppresses the effects of slow spectral fluctuations. The repeatability and the high degree of control of the experiment open the road toward deterministic fabrication of plexitonic devices at ambient conditions, providing single‐photon nonlinearities.

## 2 Results

Our experiments are based on plasmonic slit nanoresonators (PNR) fabricated at a corner of a mono‐crystalline gold microplatelet.^[^ ^20^ ^]^ Such PNRs are used as scanning probe tips in an atomic‐force microscope (AFM) setup as depicted in Figure 1A, which also includes a scanning confocal optical microscope (Figure S1A, Supporting Information). Utilizing the atomic‐force microscopy capabilities of the setup provides nanometer precision in positioning Qdots beneath the PNR tip while the confocal microscope records the luminescence of the system. With these advantages, a typical strong coupling experiment can be performed by precisely tuning the position between the PNR and the single Qdot. Additionally, it is possible to sequentially couple different single Qdots to the same PNR, as the two coupled entities can be detached by retracting the tip. Moreover, the uncoupled properties of the PNRs and Qdots, such as their resonances, can be carefully examined before and after the coupling experiment.

We use He‐ion beam milling to fabricate PNRs with suitable resonances. A scanning electron microscopy (SEM) image of a fabricated PNR probe tip is displayed in Figure 1B (More details in Figure S2, Supporting Information). Compared with our previous study,^[^ ^20^ ^]^ the PNR fabricated using the He‐ion beam milling technique exhibits a better‐defined structure with a narrower slit width, which reflects in its enhanced quality factor. For the present experiment we choose the second‐order plasmonic mode because its quadrupolar character naturally suppresses radiative losses, reflecting in a ‐ for a plasmonic resonator ‐ comparatively high Q‐factor on the order of 19. The resonances of PNRs can be tuned by varying length and width while maintaining the Q factor, as evident from finite‐difference time‐domain (FDTD) simulations in Figure S3 (Supporting Information). Q‐factors are experimentally verified by recording the shaping of the intrinsic linear photoluminescence (PL) spectrum of gold exited by a 532 nm continuous‐wave laser diode in the vicinity of the PNR. The resonance and Q factor of the PNR are extracted from such gold PL spectra using a cumulative fit that includes an exponential decay to accommodate the unshaped PL background and a superimposed Lorentzian for the PNR emission. Notably, gold PL is only visible under comparatively large excitation power (hundreds of microwatts), which is significantly higher than the one used for coupling experiments (1μW). From the PL measurement in Figure 1C, a distinct peak indicates the resonance of the PNR at approximately 1.9eV. Fitting yields a Q factor of 19, which matches well with FDTD simulations. An extended discussion about PNR slit cavities and their tunability can be found in Note S1.2 (Supporting Information).

We investigate the coupling of PNRs to single colloidal CdSe/ZnS semiconductor nanocrystals (Thermo Fisher Scientific Inc., QDot655 ITK Q21321MP) with an average emission energy of 1.893eV (655 nm, Q‐factor: 34) dispersed on a glass cover slip at a coverage of about 1 emitter/$\mu m^{2}$ by spin coating, as shown in Figure 1E. Unlike in our previous study, this experiment uses undoped colloidal CdSe/ZnS QDots, without tellurium doping, resulting in a cleaner and sharper emission spectrum—crucial for obtaining well‐resolved coupled spectra with only two distinct split peaks. In a second step, the coverslip is spin‐coated with *10* nm polymethylmethacrylate (PMMA) film to immobilize the Qdots. An atomic force microscopy (AFM) image of such a sample is shown in Figure S5a,b (Supporting Information). When analyzing the second‐order autocorrelation function *g* ^2^(τ) of the Qdots' emitted PL, a *g* ^2^(0) = 0.08 is typically obtained (Figure 1D) at zero time delay τ alongside the typical blinking behavior (Figure S5D, Supporting Information). Both observations signify the fact that a single emitter is investigated. Further details of the single‐emitter characterization are presented in Note S1.3 (Supporting Information).

After pre‐characterizing the PNR and the Qdot individually to ensure spectral overlap as well as single‐emitter character of the Qdot emission, the PNR is positioned in close proximity to the Qdot to investigate possible strong light‐matter coupling. To this end, a λ_exc_ = 532 nm laser spot (power: ≈1μW, intensity: 8.8 × 10^6^ W/m^2^) is focused at the PNR tip, and then the Qdot is moved beneath the tip and scanned precisely via a piezostage with nanometer resolution (Supplementary Note S3). Note that at such a low excitation intensity, gold PL is too weak to be detected above the background signal. However, at the selected wavelength, the light acts as an efficient non‐resonant pump of the Qdots resulting in a PL at around 655 nm, which consequently couples with the matching PNR resonance. It is important to reiterate that the detected PL spectra in our measurements, due to the low excitation intensity used, can never show two peaks due to mere combination of uncoupled Qdot and PNR, since the emission of the latter is far too weak in comparison to the Qdot emission (Figure S7).

**Figure 2:**
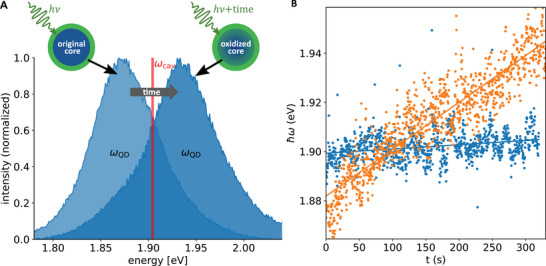
Light‐induced oxygen‐dependent Qdot blue‐shift. A) Illustration of the core size reduction due to oxidation and the resulting increased transition energy. B) Time development of the resonance maxima as a function of time obtained by fitting Lorentzians to individual Qdot photoluminescence spectra at ambient conditions in air (orange) and in argon atmosphere (blue).

To achieve a complete anticrossing of PNR and QDot, we make use of a well‐known slow, light‐induced and oxygen‐dependent blue‐shift of QDots.^[^ ^22^, ^23^ ^]^ We confirmed this effect (as shown in **Figure** **2**A) and found that it can be prevented in an oxygen‐free atmosphere, e.g., in argon, or in absence of the pump light (Figure 2B). The linear blue‐shift of a bare Qdot as a function of time in air, respectively its absence in argon atmosphere, is illustrated in Figure 2B and Figure S6 (Supporting Information). A possible explanation for the observed pronounced light‐induced blue shift in the presence of oxygen over time is the oxidation of the outer shell of the core, which effectively shrinks the core, leading to a controlled spectral shift of the Qdot PL by up to 100meV over several minutes of continuous illumination.^[^ ^23^ ^]^ Besides, as confirmed in Figure S6 (Supporting Information), the linewidths of single QDots under ambient and argon conditions are comparable, indicating that the light‐induced, oxygen‐dependent blue shift does not introduce additional decay channels for the QDot.

Within about 300 s, the Qdot in air shows a total blue‐shift of about 62.8meV, while in argon atmosphere for the same Qdot, the resonance energy remained unchanged. The blue‐shifting of the Qdot resonance is an irreversible process that continues to progress linearly with increasing measurement times and typically ends with the final photo‐bleaching of the Qdot.^[^ ^23^ ^]^

**Figure 3:**
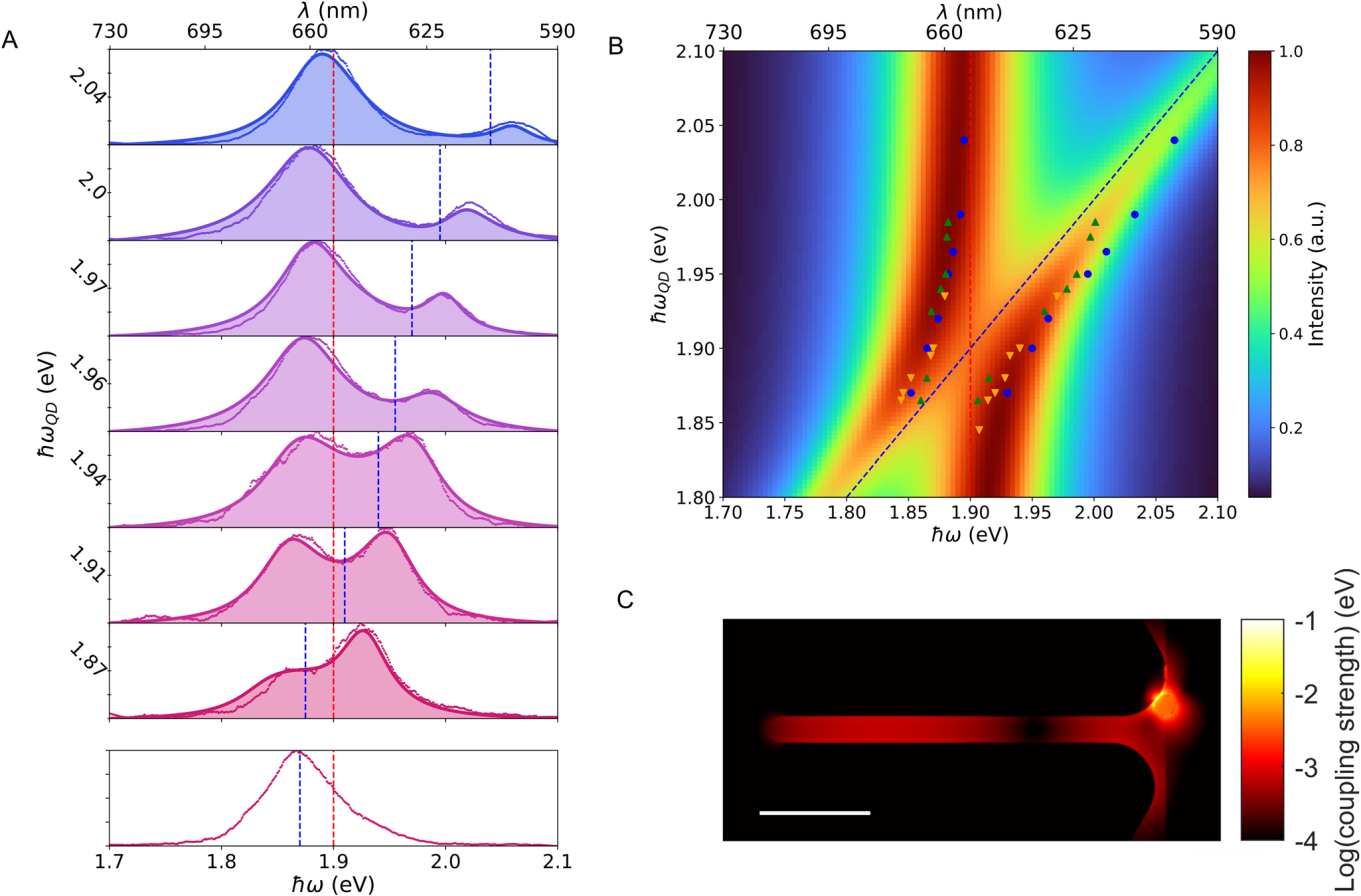
Anticrossing in a coupled Qdot‐plasmonic nanoresonator (PNR) system exhibiting strong coupling (SC). A) The bottom panel indicates the initial resonance of the uncoupled Qdot. The upper panel presents strongly coupled PL spectra (small dots) over time (bottom to top), fitted using a quantum model (solid lines). The dashed red and blue lines indicate the PNR and fitted Qdot resonances, respectively. Fitting parameters are provided in Table S1 (Supporting Information). B) Simulated anticrossing spectra of a fixed PNR resonance (ω_a_, dashed red line) and a linearly shifting Qdot resonance (ω_σ_, dashed blue line) calculated by the quantum model. The parameters used to model the anticrossing are found in Note S5 (Supporting Information). Peak positions of three SC experiments (with three different Qdots) are labeled by symbols of different shapes and colors, all exhibiting a complete and consistent anticrossing with the same PNR. The additional two datasets of anticrossing can be found in Figure S13 (Supporting Information). C) Map of the coupling strength *g* in eV (log scale) as obtained by classical electromagnetic modelling (FDTD) using a point‐like dipole moment of 5 Debye. Scale bar: 50 nm.

A resulting time series of photoluminescence spectra (from bottom to top) of a strongly‐coupled Qdot‐PNR system is shown in the upper panel of **Figure** **3**A. The only changing parameter in these spectra is the time‐dependent, oxygen‐induced blue‐shift of the Qdot emission. As a reference, the initial resonance of the uncoupled Qdot is measured (bottom panel in Figure 3A). The dashed lines indicate the measured PNR bare resonance (red) and the fitted Qdot resonance (blue), respectively, demonstrating the expected sweeping of the Qdot resonance over the PNR resonance, leading to the observed anticrossing. The spectra shown in Figure 3A are selected at approximately equidistant time intervals from a continuous measurement to ensure uniform additional energy shifts of the quantum dot (Qdot) resonance across the rectangular regions in the anticrossing dataset, as presented in Figure S16A (Supporting Information). This dataset is directly obtained from the continuous measurement depicted in Figure S15 (Supporting Information). A more detailed discussion on the selection of spectra and data processing can be found in Note S7 (Supporting Information). Fluctuations in the time‐sequenced coupled spectra are inevitable at room temperature and primarily arise from two factors. First, spectral diffusion of the Qdot contributes to these variations, as also observed in the single Qdot resonance monitoring shown in Figure S6 (Supporting Information). Second, we record all the spectra with a high time resolution of 33 ms per spectrum, which is unique and allows the observation of blinking dynamics of the coupled system in the first place. The integration time can be extended to reduce noise, as demonstrated by averaging of spectra within each dashed rectangular region in Figure S16B (Supporting Information), where the two dashed lines guide the visualization of a clearer anticrossing behavior. However, due to intrinsic spectral diffusion in Qdot emission, the averaged spectra appear broadened.

As a function of time, the strongly coupled spectra change from red‐detuning to blue‐detuning. The resulting spectra generally exhibit two peaks and can be nicely modeled by a quantum Jaynes‐Cummings model.^[^ ^24^ ^]^ This model is widely used for describing the light‐matter interaction of a single two‐level system and a single‐mode cavity. In order to model incoherent pumping, radiative decay (dissipation), and pure dephasing, we model the system via a master equation in Lindblad form. The detailed quantum model and fitting strategy are described in Note S5 and S6 (Supporting Information). The validity of this simplified description is sometimes questioned due to a possible mode overlap of neighboring modes.^[^ ^25^ ^]^ However, for the present slit design, the cross‐talk of adjacent modes is highly suppressed,^[^ ^26^ ^]^ which is also verified by our characterization of the uncoupled PNR via gold PL showing only one resonance order within the spectral range relevant for coupling (Figure 1C). Furthermore, the PNR slit modes exhibit a quadrupolar character, leading to enhanced Q‐factors of 19, mostly due to reduced radiative losses. It is therefore safe to assume that our PNR indeed exhibits only a single plasmonic mode in the coupling range. In the future, the quality factor of the slit cavity can be further improved by red‐shifting the operating wavelength to reduce Ohmic losses associated with gold absorption. Higher‐order modes, such as the octupole mode, may also provide a higher quality factor. However, a key challenge is the increased crosstalk between different mode orders due to the reduced free spectral range. A similar issue arises when using dielectric single elements (e.g., nanospheres) as cavities. While they can support higher‐order modes—such as magnetic quadrupole modes—with relatively high quality factors,^[^ ^27^ ^]^ their mode volume is considerably larger than that of the PNR which reduces the coupling strength. Furthermore, a higher Q factor of the resonator has to be matched by the linewidth of the emitter to maintain spectral overlap.

An important conclusion from our observations is that after long coupling experiments with Qdots that are subject to the oxygen‐induced blueshift, the resulting SC spectra will typically always show a split spectrum in which the Qdot is strongly blue‐detuned with respect to the PNR. This can be a surprising observation if the time‐dependent blue shift is not taken into account and the experiment is assumed to have started at zero detuning. Under such conditions, the observed splitting is far larger than the splitting at zero detuning and can lead to a significant over‐estimation of the coupling strength. Indeed, several reports demonstrated blue‐shifted strong‐coupling spectra of individual Qdots with plasmonic resonators.^[^ ^13^, ^28^, ^29^ ^]^

Time‐dependent measurements of three different Qdots with the same PNR are collected in Figure 3B. Peak maxima, ω_−_, ω_+_, resulting from fitting the experimental spectra with the quantum model, are indicated by three different symbols. A typical anticrossing map predicted by the quantum model, assuming a fixed PNR resonance (red dashed line) and a linearly detuned Qdot resonance (blue dashed line), is displayed in the background. Note that each individual Qdot produces a complete anticrossing with the PNR. All plots show excellent agreement and can be well modeled, enabling us to extract the average coupling rate at zero detuning from the quantum model as *g* = 50meV. Nonetheless, variations in coupling strength are still observed, arising from structural differences in the colloidal QDs and their orientation with respect to the electric field polarization at the PNR apex.

For a strongly coupled system, at least one Rabi oscillation cycle needs to be completed in the time domain, which requires 2*g* > (γ_a_ + γ_QD_)/2, where γ_a_ and γ_QD_ are the total decay rates of the PNR and quantum dot (including dephasing), respectively. Both parameters are extracted by fitting the FWHM of the PL curves of both uncoupled entities. The PNR has a Q‐value of about 19 because of its quadruple mode pattern. This results in $\gamma_{a}=100\;meV$ while γ_QD_ is roughly about 56meV. Our hybrid system, therefore,‐ fulfills the above criterion due to the low‐loss PNR and the high single‐emitter coupling strength.

Second‐order correlation *g* ^2^(τ) measurements have also been performed with the system being strongly coupled. However, in contrast to the clear dip below 0.5 at time‐delay τ = 0 and finite decay rate observed for the uncoupled Qdots, we were unable to detect such a dip in strong coupling. The following analysis explains why such experiments are very difficult due to fundamental reasons. When the PNR mode is strongly coupled to the Qdot, the lifetime of two (upper and lower) polaritons can be described using Hopfield coefficients ^[^ ^30^ ^]^ (Note S9, Supporting Information). The decay rate of both upper and lower polariton will be dominated by the very fast decay of the PNR mode. Specifically, when the PNR mode is on resonance with the two‐level system, the decay rate of the hybrid system can be written as (γ_*a*_ + γ_QD_)/2, which is in the femtosecond range. This renders a dip at zero delay very difficult to resolve since the time bin resolution in typical correlated photon counting experiments is limited to a few hundred picoseconds. Under such conditions, the typical pulse lengths (for example, approximately several picoseconds) used in time‐resolved experiments are long compared to the lifetime of the polaritonic states, which results in an effective cw‐type driving. In Note S9 (Supporting Information), we use a Monte Carlo method to simulate the autocorrelation under CW and pulsed excitation. As shown in Figure S20 (Supporting Information), a clear dip under SC conditions is only visible when the time resolution reaches femtoseconds.

## 3 Discussion

In the dipole approximation,^[^ ^31^ ^]^ the coupling strength g(r) can be estimated as $g\left(r\right)=\mu \sqrt{\frac{\hbar \omega }{2\varepsilon_{0}V\left(r\right)}}$, where μ is the dipole moment of the quantum dot and V(r) is the effective mode volume of the PNR's resonant mode at position r. FDTD simulations have been performed to accurately determine the mode volume based on quasi‐normal‐mode theory^[^ ^32^ ^]^ (Note S4, Supporting Information). Also, the finite size of the Qdot is considered by modelling it as a sphere of semiconductor with Lorenzian permittivity (see Note S4, Supporting Information) to mimic its dipolar two‐level transition.^[^ ^5^ ^]^ Based on uncoupled Qdot lifetime measurement, a dipole moment of 5 Debye is assigned to the Qdot in the simulated coupling‐strength map displayed in Figure 3C. Inspection shows that the coupling strength *g* indeed reaches up to 50meV in agreement with our experimental results when the PNR is in close proximity to the Qdot. The variance of the coupling‐strength map also indicates the fact that near‐field local effects greatly modify the coupling behavior, especially in the close proximity regime.^[^ ^20^ ^]^ It is also possible to check the presence of strong coupling in our system by performing both classical time‐ and frequency domain FDTD simulations (Note S4, Supporting Information). As expected intuitively, the spectral splitting as well as the corresponding energy exchange in the time domain between the PNR and the Qdot can be observed (Figure S8, Supporting Information). Both of these studies reveal a coupling strength compatible with our experiments and the quantum model.

In summary, we have experimentally demonstrated strong coupling of a PNR scanning probe precisely positioned with respect to a single Qdot at ambient conditions. Our findings provide compelling evidence of strong coupling, as we not only observe the typical energy splitting in the spectral domain but also a complete anticrossing map in PL spectra consistent with a quantum model. Anticrossing was achieved by utilizing the light‐induced oxygen‐dependent blue‐shift in the photoluminescence of core/shell Qdots. Our scanning approach also enables us to carefully check each uncoupled partner before and after the coupling experiment. Our experimental results are consistent with the Jaynes‐Cummings model, which allows us to extract the coupling spectra and determine a mean coupling energy of 50meV by fitting experimental spectra, and consistent with classical field simulations as to be expected in the single‐excitation regime. Our findings take us another step forward toward using strong light‐matter coupling as a resource in quantum information and quantum sensing schemes, as well as toward exploiting the single‐photon nonlinearity of the underlying Jaynes‐Cummings model even at room temperature. Especially, tunable anticrossing dynamics offer promising avenues for exploring novel physical phenomena, such as the dark state in a single‐slit cavity coupled to multiple tunable quantum dots.

## Conflict of Interest

The authors declare no conflict of interest.

## Supporting information

Supporting Information

## Data Availability

The data that support the findings of this study are available in the supplementary material of this article.
