# Supplementary material for: Anticrossing of A Plasmonic Nanoresonator Mode and A Single Quantum Dot at Room Temperature
Source: Adv Sci (Weinh). 2025 Aug 25;12(36):e06676. doi: 10.1002/advs.202506676 (PMC12462928; doi:10.1002/advs.202506676)
Supplement: Supplementary file 1 — Supporting Information [file ADVS-12-e06676-s001.pdf]

ADVANCED SCIENCE
Open Access

Supporting Information

for *Adv. Sci.*, DOI 10.1002/advs.202506676

Anticrossing of A Plasmonic Nanoresonator Mode and A Single Quantum Dot at Room Temperature

Daniel Friedrich, Jin Qin, Benedikt Schurr, Tommaso Tufarelli, Heiko Groß and Bert Hecht**

Supporting Information

Title: Anticrossing of A Plasmonic Nanoresonator Mode and A Single Quantum Dot at Room Temperature

Daniel Friedrich, Jin Qin, Benedikt Schurr, Tommaso Tufarelli, Heiko Groß, and Bert Hecht**

D. Friedrich, J. Qin, B. Schurr, H. Groß, B. Hecht
Address: Nano-Optics and Biophotonics Group, Experimentelle Physik 5, Physikalisches Institut, Universität Würzburg, Würzburg & D-97074, Germany.
Email Address: jin.qin@uni-wuerzburg.de; hecht@physik.uni-wuerzburg.de
T. Tufarelli
Address: Independent Researcher, Beeston, NG9, United Kingdom

S1. Characterization

S1.1 Setup

The PL measurement setup is sketched in Figure S1. A 532nm continuous wave laser (AIST-NT ROU006) is used as excitation source which is focused by a high numerical aperture objective (Nikon CFI P-Apo 100x, NA 1.45). Typically, a power of $1\mu W$ is used to characterize single Qdot emission and during coupling experiments, whereas $500\mu W$ is applied to examine the gold PL. The recorded PL signal passes through a dichroic mirror and is directed to a spectrometer (HORIBA iHR320) with an electron-multiplied, charge-coupled device (Andor Newton 970p EMCCD). The tip position is controlled by a stepper motor for coarse alignment. The sample mounting stage and objective are controlled by two independent piezoelectric stages, allowing the optimization of the relative positions of tip and Qdots. Details of hyperspectral PL maps obtained in our setup are discussed in Supplementary Note S3. Time-resolved photon statistics measurements are performed using a time-correlated single-photon counting (TCSPC) setup. The PL signal is split by a 50:50 beam splitter and recorded with two single-photon counting avalanche photodiodes (APDs, SPCM-AQR). Photon arrival events are counted and timed by a field-programmable gate array (FPGA) (qutools quTAU H+).

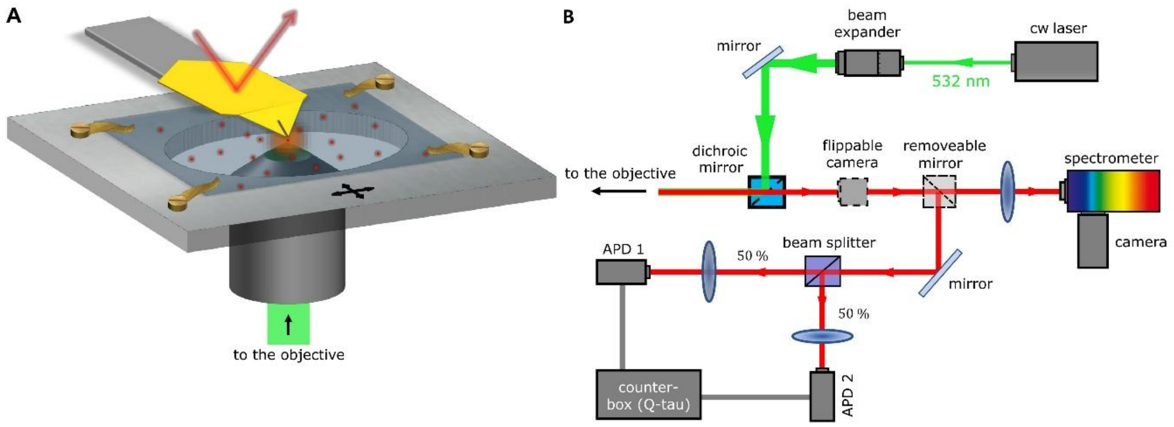

Figure S1 Setup for strong coupling measurements. A) Scheme of scanning probe setup. B) The optical setup of photoluminescence and photon statistic measurements. A 532nm continuous wave laser (green lines) is sent through a beam expander onto a dichroic mirror and into a microscope objective which focuses the laser light onto the sample surface into a diffraction-limited spot and excites either the quantum dots alone or the hybrid system consisting of emitter and plasmonic nanoresonator (PNR). The emitted photoluminescence (red lines) is collected by the microscope objective and transmitted towards the dichroic mirror (Semrock HC BS R532 1 lambda PV flat). A flippable camera allows us to observe the sample in the focal plane of the objective and the approach of the PNR scanning probe

towards the sample. After passing the dichroic mirror, a removeable mirror guides the signal either into the spectrometer or towards two APDs. The spectrometer performs a spectral analysis of the emitted signal, whereas the APDs provide temporal information about photon arrival times needed for photon statistics. To this end the two APDs are connected to a photon-analysis counter box (quTau Time-to-Digital Converter) which is controlled and read out by a computer.

S1.2 Plasmonic nanoresonator

To fabricate the PNR we are using gold microplatelets synthesized in solution based on the recipe described in literature[1–3]. Typical microplatelets exhibit a characteristic extension of about 60 μm and a thickness of around 60 nm. A suitable platelet is transferred on the top end of a contact mode AFM cantilever (doped silicon and no reflective coating, CONT-50, NanoWorld Pointprobe, NanoAndMore GmbH), with one corner extending 5 μm-10 μm beyond the cantilever (see Figure S2A). The original silicon tip of the cantilever needs to be removed by gallium fibbing before hand. After fabricating the PNR slit by means of a helium-ion milling (Orion nanoFab, Zeiss), the corner is bend down by low-dose ion irradiation beneath the corner to induce a folding (see Figure S2B)[4]. A scanning electron microscopy image of the fabricated PNR is displayed in Figure S2C. The resonance of the PNR can be tuned to match the Qdot resonance of 650 nm by adjusting the slit's length and width. The design parameters are optimized by finite-difference time-domain (FDTD Solutions, Lumerical) simulations and suggest that a 210 nm long and 15 nm wide slit should match the Qdot emission at 650 nm.

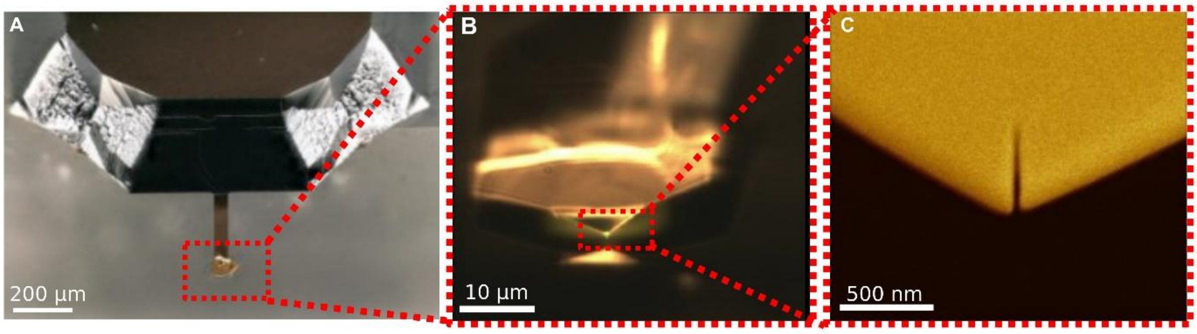

Figure S2 Plasmonic nanoresonator. A) and B) optical microscope images of the fabricated PNR on top of an AFM cantilever. C) SEM image (tilted view) of the PNR with a 210 nm long and 15 nm wide slit.

FDTD simulations also yield the Q-factors of the PNR resonances. To this end, a broadband electric dipole source is placed at the gap center of the PNR to excite modes of different orders. As we are interested in the second-order resonance, a dipole position along the PNR slit is chosen close to the expected antinode of the mode profile. Far-field emission is collected by recording the emitted power. Suitable temporal apodization is used to suppress dipole source contributions in the mode's field profile. Due to the quadrupolar character of the second-order mode, which minimizes radiative losses, a Q-factor of 20 is extracted by fitting the resulting far-field spectrum with a Lorentzian, supporting our experimental findings reported in the main text. The PNR resonance can be tuned by changing the slit width and length. Typically, with a larger slit length and width, the second-order resonance of the PNR red-shifts and narrows. In Figure S3, the changes in resonance and FWHM of the 2nd mode are plotted as a function of the slit length ranging from 180 nm to 380 nm at a fixed slit width of 15 nm. Due to the quadrupolar mode pattern a high Q-factor is always maintained.

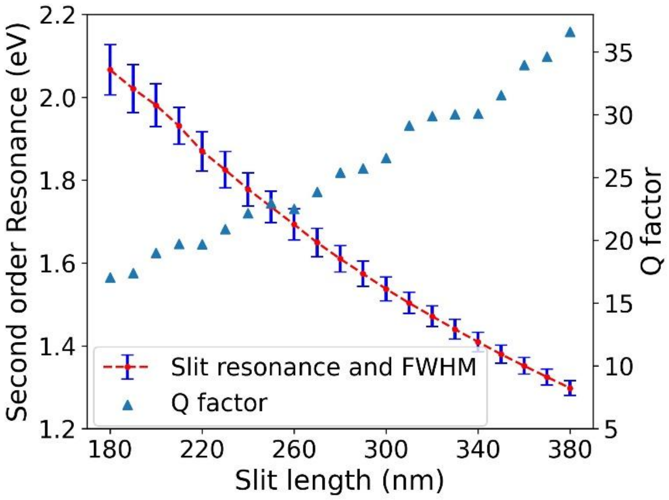

Figure S3 Slit cavity resonance and Q factor with different slit lengths. Slit-length dependence of the second-order resonance of a PNR (red-dashed line) covering slit lengths from 180 nm to 380 nm (left axis). The error bars indicate the full width at half maximum (FWHM) of the resonances. The blue triangles indicate the corresponding Q-factors (right axis).

The spectrum of the PNR is measured via the shaping of the intrinsic linear photoluminescence (PL) of gold exited by a 532 nm continuous wave laser diode ($4.4 \times 10^9\ W/m^2$) in the vicinity of the PNR. Benefiting from the local field enhancement of the plasmonic mode in PNR and the correspondingly increased LDOS, the PL background exhibits a peak-like feature which is visible above the noise for sufficiently high pump

powers. The resonance energy and Q factor of the PNR are extracted from the gold PL spectrum using a cumulative fit which contains an exponential decay to accommodate the unstructured background and a Lorentzian for the PNR emission peak:

$$f(\omega) = \frac{A_a e^{-b\omega}}{\gamma_a[1+4(\omega-\omega_a)^2/\gamma_a^2]} + A_a e^{-b\omega} + c \qquad \text{(S1)}$$

where A_a, γ_a, ω_a indicate the amplitude, FWHM, and resonance of the PNR, respectively. The resulting spectrum is shown in Figure S4A-D for the PNR probe used in the reported experiments.

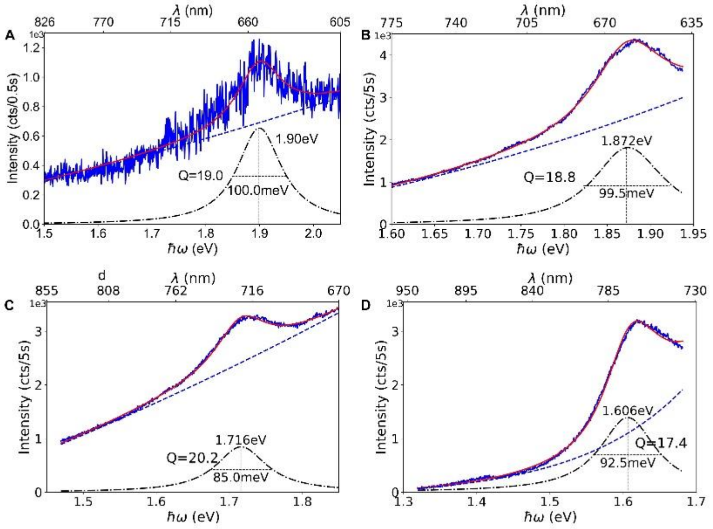

Figure S4 Characterization of plasmonic nanoresonators with different lengths via gold PL. The blue solid lines represent the measured PL spectra which clearly show shaping of the gold PL background due to the presence of the resonator for different slit lengths. Spectra in A-D) are fitted with a Lorentzian on top of a background that decays exponentially towards lower energy (red line) Equation S1.

Experimental PL spectra of different slit lengths (different resonances) are displayed in Figure S4A-D together with the corresponding fits. The expected slit-length dependence of the resonance is faithfully recovered, along with the comparatively high Q factors.

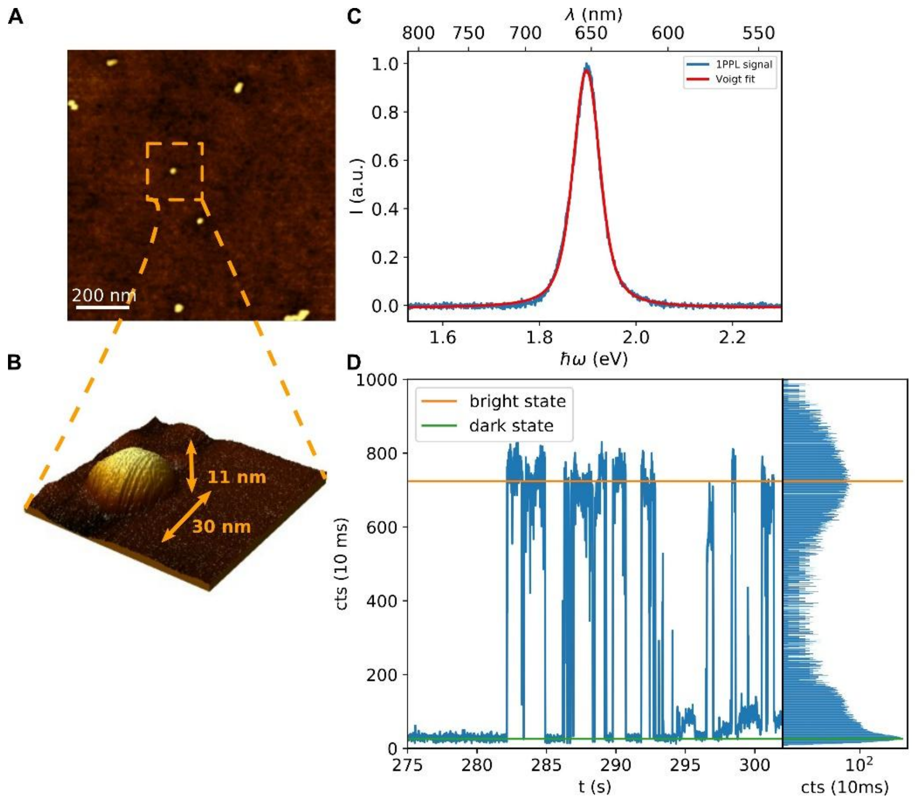

Figure S5 Characterization of a single Qdot. A) AFM-scan of a randomly picked area on a glass substrate covered with quantum dots without PMMA layer. Single Qdots as well as smaller agglomerates of nanocrystals (clusters) are distributed randomly after spin-coating. For measurements of strong coupling, the separation between single particles is decisive to perform strong-coupling experiments with single Qdots. A 3D representation of a single Qdot is depicted from the AFM-image in B). It shows the detailed shape and dimensions of an exemplary Qdot. C) provides an exemplary PL-spectrum of a single Qdot embedded in a PMMA layer on a glass substrate. The Qdot exhibits a stable peak position at around 655nm. In D), a typical recorded time-trace of the Qdot's emitted signal is depicted, providing statistical information about the blinking characteristics of the Qdot (see histogram). For better visualization, just a small excerpt of a longer time-trace is displayed. The histogram on the right indicates a two-state blinking behaviour (bright and dark state).

S1.3 Quantum dots

For all measurements, we use commercial colloidal semiconductor core-shell quantum dots (Qdot 655 ITK Carboxyl Quantum Dots Q21321MP, Thermo Fisher Scientific Inc.). The quantum dots consists of a CdSe core and a ZnS shell[5]. The shell to some degree suppresses light-induced spectral diffusion and photo-bleaching as well as surface trap states. To fabricate samples in which single quantum dots are sufficiently separated, an aqueous solution

of CdSe/ZnS Qdots is first spin-coated on the microscope coverslips (Gerhard Menzel GmbH), which are cleaned in acetone and ethanol using an ultrasonic cleaner. In a second step, a thin (10 nm) PMMA film (0.4% PMMA dissolved in anisole by weight)is spin-coated to prevent the quantum dots from being picked-up or being pushed by the scanning PNR-probe. In a typical sample, a lateral separation of at least $1\mu m$ between different Qdots is obtained which guarantees that only one single quantum dot couples to the PNR probe at a time. To characterize Qdot samples, we use a combination of atomic force microscopy (AFM) and confocal microscopy. In Figure S5A, an AFM-scan is displayed showing the typical lateral distribution of single quantum dots and smaller agglomerates on a clean glass substrate prior to PMMA-coating. A 3D representation of a single quantum dot, marked in the 2D AFM-scan, is displayed in Figure S5B.

Photoluminescence measurements of single Qdots at room temperature excited at a wavelength of 532 nm show a narrow emission peak at around 655 nm (Figure S5C). The emission spectrum of the nanocrystals is stable in time showing no significant spectral fluctuations. A typical time-trace of a quantum dot's PL intensity during permanent excitation recorded by two single-photon counting APDs is presented in Figure S5D. The time trace exhibits typical blinking behavior with a dark and a bright state, as well as the corresponding intensity histogram. In our previous study[4], we used CdSeTe/ZnS core/shell nanocrystals as the quantum emitter, where we observed four split peaks when it was strongly coupled with the slit cavity. We explained that the outer two peaks resulted from strong coupling of the charged Qdot state. We suggested that in strong-coupling, the system provides a fast radiative decay channel that surpasses Auger recombination and therefore renders charged Qdot excitations optically bright. In this study, we use a different quantum emitter, CdSe/ZnS, which features a smaller core/shell size and lacks tellurium doping. We therefore expect the Auger decay rate to be even faster due to the reduced volume. If the Auger decay rate is comparable to or faster than the radiative decay rate under strong coupling, the charged Qdots will remain optically dark, allowing only the neutral excitons to contribute to the coupling behavior.

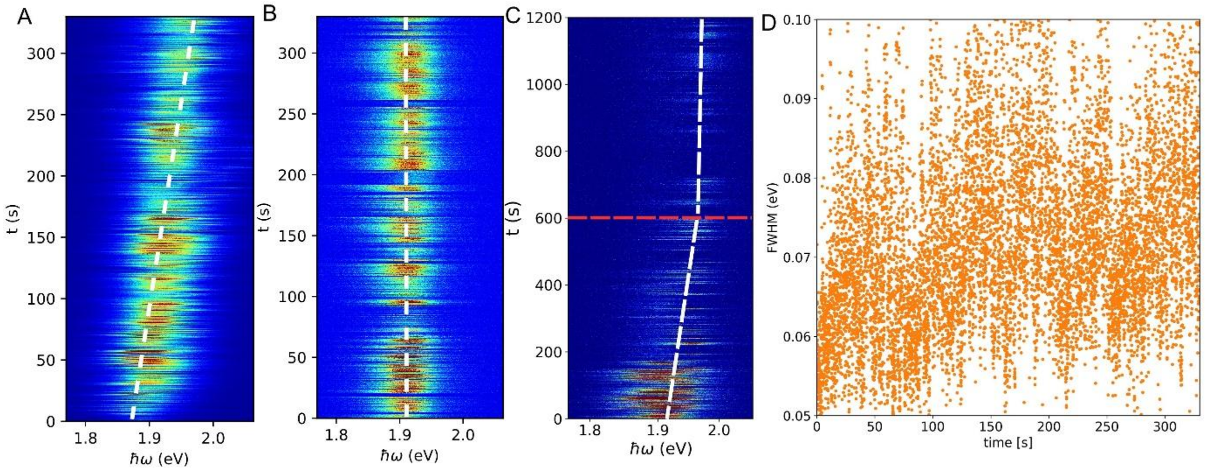

Figure S6 Time traces of single-quantum dot PL spectra at different conditions. A) The single Qdot is placed in ambient condition and B) in argon atmosphere. C) The single Qdot is first exposed under ambient condition (from 0-600s), during which a characteristic oxygen-induced blue shift is observed. After 600s, argon gas is introduced, effectively halting the blue shift and stabilizing the Qdot's transition energy (indicated by the white dashed line). All the PL spectra of single Qdot emission are taken under the lase power of $1\mu W$. D) The linewidth of single Qdot emission extracted from A).

S2. Qdot blue-shift

A light-induced oxygen-dependent blue-shift of colloidal CdSe/ZnS Qdots was reported in 1996[6]. To confirm such a light-induced oxygen-dependent blue shift in our Qdots we show the PL spectrum of an exemplary individual Qdot recorded over a time span of several minutes during which a spectrum has been recorded every 33 ms to trace the spectral changes. The resulting spectra are plotted in Figure S6A as a map with time increasing from bottom to top and a color-coded PL intensity. A significant blue-shift of about 30 nm can be observed over time in agreement with earlier observations[7]. To further proof the oxygen dependence we perform the same measurement in an argon atmosphere (see Figure S6B). In absence of oxygen, the Qdot resonance does not shift over time thus confirming the oxygen dependence of the process. For a better visualization we fitted all spectra in Figure S6A and B with an Lorentzian function and plot the peak positions in Figure 2B. A linear blue shift with time is observed. Besides this, we also extract the emission linewidths of blue shifted Qdot in Figure S6A, as shown in Figure S6D. The linewidths exhibit only a slight broadening trend over time, while still remaining within the range of typical random fluctuations.

To verify the tuning capability of the light-induced, oxygen-dependent effect, we present the results in Figure S6C. Initially, the Qdot is exposed to ambient air (0–600 s), during which its emission undergoes a characteristic blue shift due to oxygen interaction. After this period, an argon gas flow is introduced to displace the surrounding oxygen. From that point onward (after 600 s), the Qdot emission stabilizes, and no further blue shift is observed. This method provides a simple and controllable way to fine-tune the Qdot emission.

A possible reason for the observed blue shift likely is oxygen diffusion in the Qdot shell which subsequently oxidizes the Qdot core. The resulting smaller effective core radius then leads to a higher transition energy[6,7]. The effect is illustrated in Figure 2A. We find that the light-induced oxygen-dependent blue-shift effect displayed in Figure S6A continues to increase linearly and can be monitored until the quantum dot is photo-bleached.

S3. PL map

Benefiting from the confocal microscope capabilities of the setup, we are able to resolve the PL of individual Qdots on the sample with high resolution. When the PNR tip lands on the sample and the microscope objective scans across it with high excitation power, the PNR resonance can be distinguished. This allows us to spatially overlap the PNR with a chosen single Qdot. Corresponding hyper PL maps, integrating different spectral ranges are shown in Figure S7A. We integrate the spectrum around the Qdots emission (1.82eV-2.0eV), resulting in an almost completely saturated image due to the high excitation power ($500\mu W$). In Figure S7B, we integrate the higher-energy photons (2.23eV-2.29eV) taking advantage of the decaying tail of the gold PL background signal. This enables us to resolve the position of the PNR. At this position, the excitation laser is perfectly aligned with the PNR tip. We then scan the Qdots sample by moving the mounting stage, driven by a piezo stage with nanometer resolution, to adjust the position of a single Qdot.

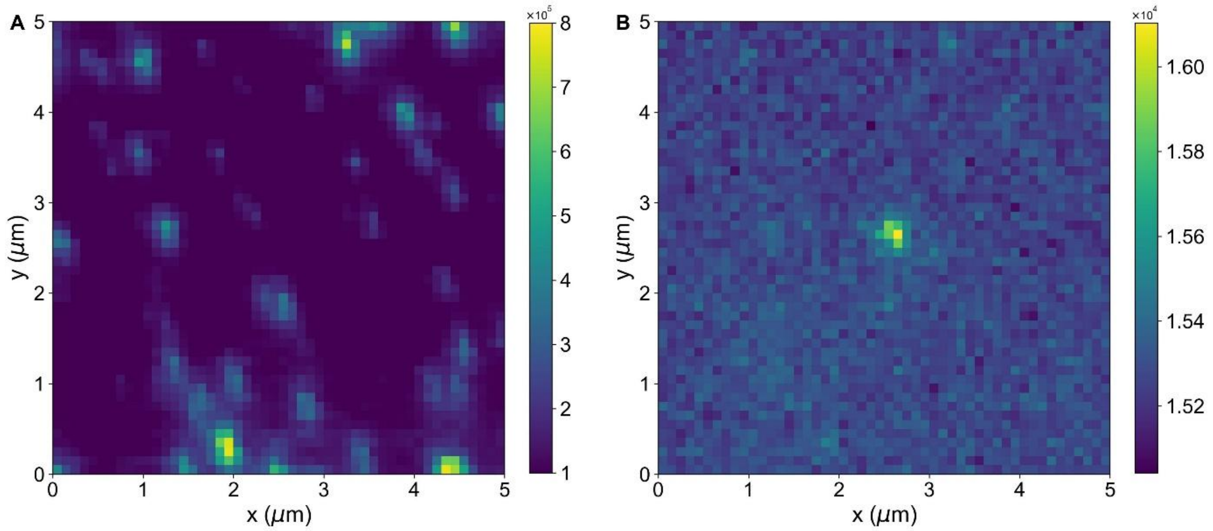

Figure S7 A hyperspectral PL map is captured when a tip with PNR is landed on the Qdots sample. A) shows the image with an integration range around the Qdots emission (1.82eV-2.0eV). B) displays the image with an integration range of high-energy photons (2.23eV-2.29eV). Note that A) and B) stem from the same hyperspectral image, integrated over different energy ranges.

S4. Classical model simulations

FDTD simulations were conducted to investigate the coupled states between a PNR and a single quantum emitter. The dielectric function of single-crystalline gold was obtained from Olmon et al [8]. To account for the quantum dot exciton state, a Lorentzian function was used to describe its permittivity: $\varepsilon_{Qdot}(\omega) = \varepsilon_\infty + f\omega_0^2/(\omega_0^2 - \omega^2 - i\gamma_0\omega)$ [9]. Here, ε_∞ is the high-frequency component of the CdSe/ZnS quantum dot matrix dielectric function, with a value of 5. The oscillator strength was set to 0.3 and the lowest transition between the exciton state and ground state was at 1.89 eV. The linewidth of the exciton state was 57 meV. The PNR geometry was optimized based on the SEM image, including rounded corners and edges. By fitting the far-field scattering spectrum with a Lorentzian function, the resonance of the PNR was found to be 1.9 eV, with a cavity decay rate of 100 meV.

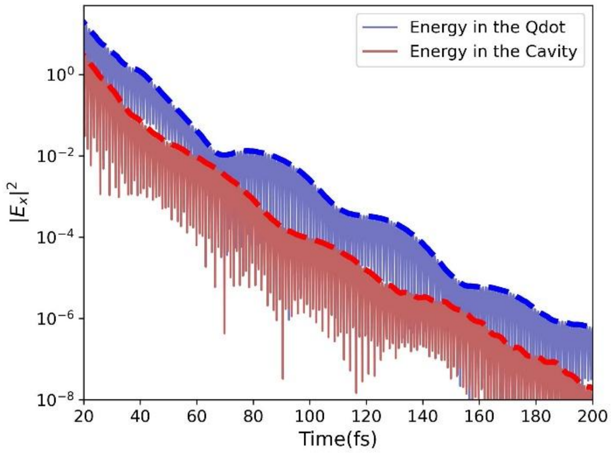

Figure S8 Evolution of time domain signals inside the slit cavity and quantum dot, respectively. Envelopes (blue and red dash lines) indicate the energy exchange between two systems. The fast modulation signals in the time trace corresponds to the intrinsic frequency of the plasmonic mode[10].

S4.1 Time-domain simulations

A characteristic behaviour of strongly coupled systems, such as two coupled oscillators, is their exchange of energy in the time domain after one emitter is excited. For example, in the case of two strongly-coupled gold nanorods with a certain gap, bonding and anti-bonding modes occur in the spectra, but energy exchange also occurs in the time domain. To observe such exchange of energy can serve as the first checkpoint in simulations to confirm that the quantum dot and PNR are indeed strongly coupled. To simulate this, the quantum dot's with its Lorentzian permittivity is placed close to the PNR, and the system is excited by an x-polarized plane wave with a pulse duration of about 18 fs. Temporal dynamics are recorded from two monitors placed inside the quantum dot and near the PNR, and the x-component of the electrical field $|E_x|^2$ is filtered to eliminate irrelevant signals. After the direct influence of the excitation pulse has vanished (at approximately 50 fs), the energy exchange pattern indeed starts to appear in the time domain signals. Due to the intrinsic losses in this coupled system, as expected, the energy exchange is visible only for a few oscillations. From the period of this oscillation (approximately 43 fs), a Rabi energy of ~98 meV can be extracted, which is close to the splitting energy observed in our experiments. This kind of time domain simulation is very helpful for validating the model and for obtaining a rough classical picture of strong coupling.

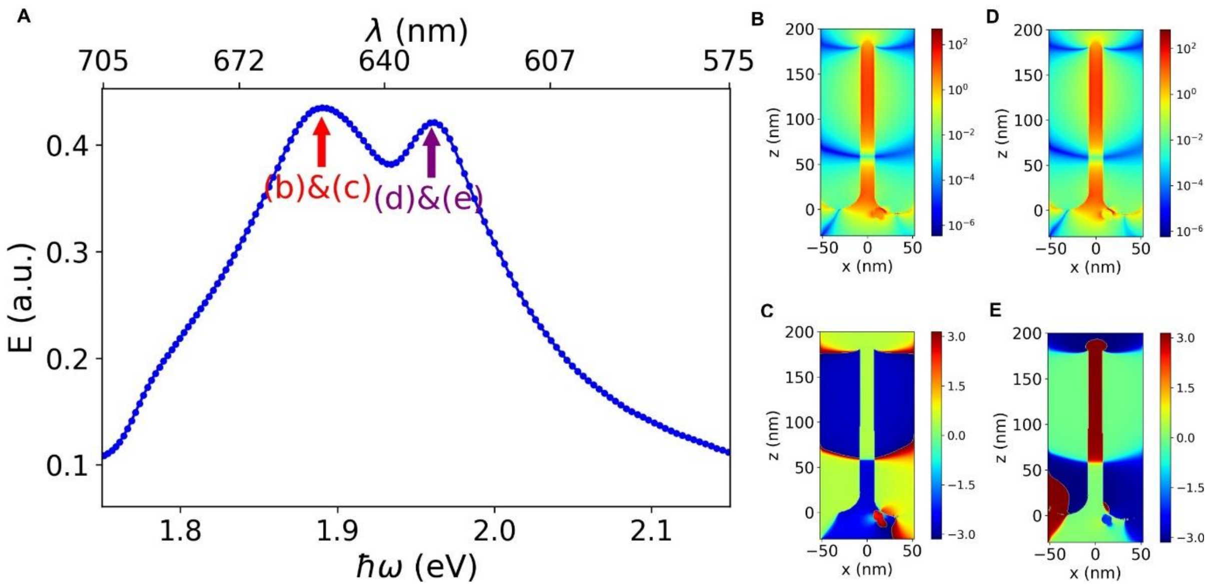

Figure S9 Classical mode patterns simulations. A) Spilt spectra obtained from the FDTD simulations including the quantum dot with a Lorentzian model permittivity. Electric field intensity B) and D) and phase C) and E) at two peaks are extracted indicating two different modes are formed.

S4.2 Frequency-domain simulations

Another feature of strongly coupled systems is the typical splitting in the frequency domain. Using classical simulations, of course only the linear behavior of the system can be obtained - the single-photon nonlinearity can only be obtained via a full quantum model. By using a Lorentzian model for the Qdot permittivity to mimic the excitation state the coupling spectrum can be extracted by detecting the electrical field amplitude in the center of the PNR. It is critical, though, to consider suitable temporal apodization to suppress the contribution of the excitation pulse. Our simulation results are presented in Figure S9A. Two new peaks (ω_- 1.88eV, ω_+ 1.96eV) with a splitting of about 80 meV occur when the resonance of both PNR and quantum dot (ω_{PNR},ω_{QD}) are set to 1.9 eV. Additionally, mode profiles recorded at the central frequency of these two peaks ($\omega_\pm$) can be obtained and the intensity and phase of the respective near-fields $|E_x|$ can be investigated, as displayed in Figure S9B-D. The near-field profile at ω_- is similar to the second-order mode of the bare PNR. In contrast, the near-field profile at ω_+ shows large field enhancement in the Qdot region. In addition, a typical phase shift occurs, indicating that a new mode is formed due to strong coupling. The simulated spectrum resembles our experimental spectra for the case of zero detuning between PNR and Qdot.

S4.3 Estimation of the coupling strength

The coupling energy between the PNR and quantum dot $\boldsymbol{g}$ is determined by the scalar product of the dipole moment $\boldsymbol{\mu}$ of the quantum dot with the vacuum field amplitude E_0 at position $\boldsymbol{r}$. $E_0(\boldsymbol{r})$ is defined as:

$$E_0(\boldsymbol{r}) = \sqrt{\frac{\hbar\omega}{2\varepsilon_0 V_{eff}(\boldsymbol{r})}} \quad \text{S2}$$

where $\hbar\omega$ is the photon energy, ε_0 is the permittivity of free space, and $V_{eff}(\boldsymbol{r})$ is the effective cavity mode volume of the PNR. Equation S2 shows that $\boldsymbol{g}$ scales with $\sqrt{1/V_{eff}}$. One possible way to estimate the coupling energy therefore is to calculate the effective mode volume. However, plasmonic cavities often have low Q factors, causing integral divergence when using the common normal mode prescription. To address this issue, we use quasi-normal modes (QNM) with complex frequencies to determine the effective mode volume, following Sauvan et al.[11]. The effective mode volume is expressed as:

$$V_{eff} = \frac{\int (\vec{E} \cdot \frac{\partial(\omega\varepsilon(r))}{\partial\omega}\vec{E} - \vec{H}\frac{\partial(\omega\mu(r))}{\partial\omega}\vec{H}) d^3\boldsymbol{r}}{2\varepsilon_0\varepsilon \max(|\vec{E}|^2)} \quad \text{S3}$$

where ω becomes a complex number whose imaginary part is determined from $Q = -\Re(\omega)/2\Im(\omega)$, $\varepsilon(\boldsymbol{r})$ and $\mu(\boldsymbol{r})$ are the relative permittivity and permeability of the simulation area, and $\vec{E}$ and $\vec{H}$ the electric and magnetic field distributions inside the PNR under x-polarized plane wave excitation. The position dependent $V_{eff}(\boldsymbol{r})$ can be rescaled by the unit less factor depending on the local electrical amplitude and permittivity. The simulation configuration is shown in Figure S10. Apodization was applied according to Ge et al.[12] The simulation area is defined by a homogeneous mesh with size of 0.5 nm.

To determine the dipole moment, we use the relation between the dipole moment and the oscillator strength of the exciton transition, which is given by $\boldsymbol{\mu} = \sqrt{\frac{f\hbar}{2m_e\omega}}$, where m_e is the electron mass. In our case, only the lowest optical transition is considered for strong coupling behaviour and its oscillator strength is determined either via lifetime or absorption measurements. Here, we measure the fluorescence lifetime τ to deduce the oscillator strength by[13,14]:

$$f = \frac{6m_e\varepsilon_0\pi c^3}{q^2 n\omega^2\tau} \quad \text{S4}$$

where m_e and q is the electron mass and charge respectively, ε_0 is the vacuum permittivity, c is the speed of light in the vacuum, n is the refractive index of the surrounding medium. Fitting the $g^2(\tau)$ measurement yields a lifetime of the quantum dot of 43.8 ns. Therefore, a dipole moment of 5 Debye is assigned to estimate the coupling energy. To determine the position-dependent coupling strength in Figure 3C, we multiply the effective mode volume by a dimensionless overlap factor, defined as $\eta = \max\left(|\vec{E}|^2\right)/|\vec{E}|^2$.

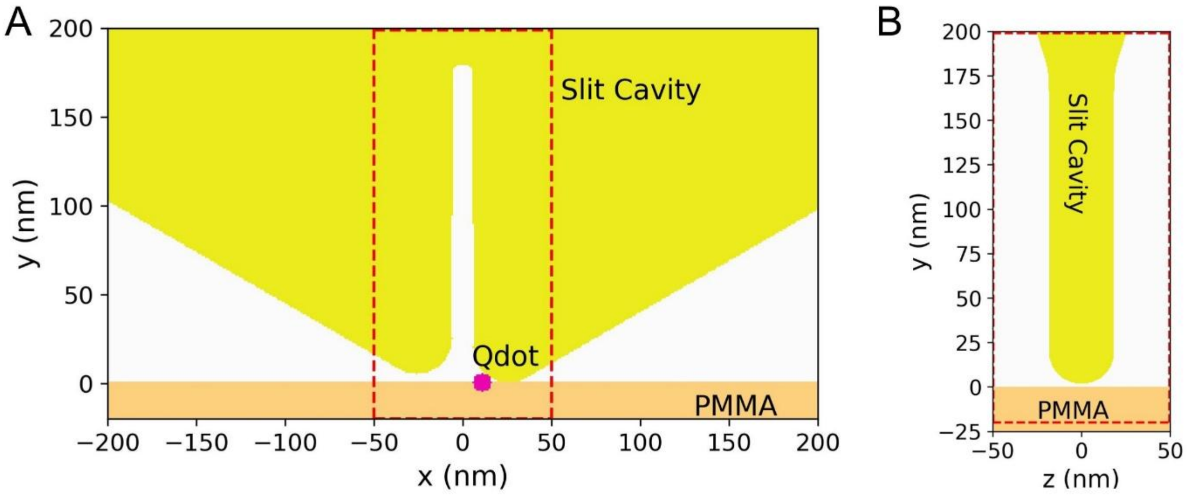

Figure S10 Sketch of the simulation volume in FDTD in a 2D projection showing the PNR (yellow), the Qdot (pink) and the glass substrate (orange). The red-dashed rectangle indicates the region of interest in our model. A), The xy plane and B) yz plane. The yz cut plane is taken at x = 30 nm.

S5 Quantum model of strong coupling

Light-matter strong coupling can be described by the Jaynes-Cummings Hamiltonian ($\hbar = 1$) [15]:

$$H = \omega_a a^\dagger a + \omega_\sigma \sigma^\dagger \sigma + g(a^\dagger \sigma + \sigma^\dagger a) \qquad \text{S5}$$

where a ($a^\dagger$) denote annihilation (creation) operators of a single Bosonic mode with the energy of ω_a, σ ($\sigma^\dagger$) represents the raising (lowering) operators of a two-level system with transition energy ω_σ. $\sigma_{x,y,z}$ are Pauli matrices. These two systems (the cavity and the quantum dot) are coupled with a coupling strength g. To obtain the spectrum of H, the dynamics of this hybrid system are studied by solving the master equation:

$$\frac{d}{dt}\rho = -i[H,\rho] + \sum_i \mathcal{L}_i(\rho) \qquad \text{S6}$$

where ρ denotes the density matrix, and $\mathcal{L}_i$ the Lindblad superoperators accounting for all kinds of dissipative contributions of the dynamics, including radiative decay ($\gamma_{a,\sigma}$), incoherent

pumping ($P_{a,\sigma}$), and pure dephasing (γ_ϕ). The sketch in Figure S11 illustrates the detailed physical meaning of each parameter. The Lindblad operators have the following form[16,17]:

$$\sum_i \mathcal{L}_i(\rho) = \sum_{c=a,\sigma} \frac{\gamma_c}{2}(2c\rho c^\dagger - \{c^\dagger c, \rho\}) + \sum_{c=a,\sigma} \frac{P_c}{2}(2c^\dagger \rho c - \{cc^\dagger, \rho\}) + \gamma_\phi(\sigma_z \rho \sigma_z - \rho)$$

S7

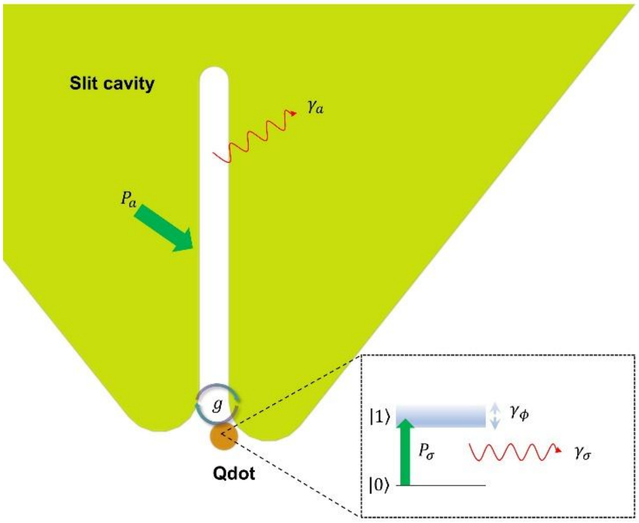

Figure S11 Illustration of the coupling behaviour between slit cavity and Quantum dots. The slit cavity is coupled with a quantum dot (Qdot) by a strength g. For the slit cavity, we consider the incoherent pump rate P_a and dissipative rate γ_a. A two-level system is used to mimic the dynamics of Qdot as (inset). Similarly, the incoherent pump rate P_σ and radiavtive loss rate γ_σ are accounted for the model. Also, the dephasing rate γ_ϕ is included.

Here, we assume the cavity emission is dominant in the hybrid system and the direct emission of the quantum dot is neglected [18]. Under these assumptions, the emission spectrum $S(\omega)$ is obtained as:

$$S(\omega) \propto \Re(\int_0^\infty \langle a^\dagger(\tau) a(0)\rangle e^{-i\omega\tau}\, d\tau) \qquad \text{S8}$$

The numerical simulations are performed by using the Python module Quantum Box (Qutip) [19]. The parameters used for generating the anticrossing map Figure 3B are $g = 50meV$, $\omega_a = 1.9eV$, $\gamma_a = 100meV$, $P_a = 3.2meV$, $\gamma_\sigma = 15neV$, $\gamma_\phi = 55.7meV$, $P_\sigma = 1.6meV$. The data in the map is normalized row by row.

S6. Fitting of the coupled spectra

In our experiments, we utilize a green laser to incoherently excite the quantum dot, hence only the incoherent pumping of the quantum dot P_σ term should be taken into consideration during

fitting. Nevertheless, some studies have shown that the cavity pumping has non-negligible photonic contributions to the overall spectrum[18]. Therefore, we consider both of these two incoherent pumping terms in our fitting. The spectra displayed in Figure 3A were processed using the Savitzky-Golay filtering[20]. As shown in Figure S12, both the original spectra and the smoothed data processed by two different algorithms—Fast Fourier Transform (FFT) and the Savitzky-Golay filtering—are displayed. We observed that the FFT-filtered data retained some high-frequency noise, but the Savitzky-Golay filtering produced a significantly cleaner result. Subsequently, the spectra are normalized between 0 and 1.

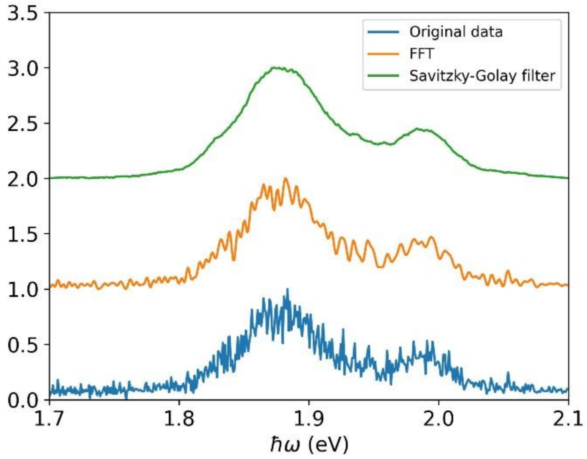

Figure S12 The original spectrum and the processed using Fast Fourier Transform (FFT) and the Savitzky-Golay filter.

The fitting approach is such that only the quantum dot resonance ω_σ and the cavity (quantum dot) incoherent pumping rates $P_{a,\sigma}$ are treated as free parameters, whereas the coupling strength g is optimized but kept constant for all the spectra. The cavity resonance ω_a ($1.91eV$) and the cavity decay rate γ_a (100 meV) are also kept fixed. They are extracted from the PL spectrum of the bare PNR. It is important to correctly describe the incoherent processes affecting the QDot. Typically, the total decay rate γ_{QD}, which is obtained from the experimentally observed linewidth of the bare Qdot emission, is composed of the radiative decay described by γ_σ and pure dephasing rate, γ_ϕ, as $\gamma_{QD} = \frac{\gamma_\sigma}{2} + \gamma_\phi$. The radiative decay rate γ_σ (15 neV) is extracted from the radiative lifetime τ (43.8 ns) of the bare Qdot using $\gamma_\sigma \tau = \hbar$. It is evident that the pure dephasing mechanism is the dominant mechanism that contributes to the linewidth of a QDot. Our previous study shows that the Qdot linewidth would be slightly affected by the presence of the gold tip [4]. Typically, the fitting process makes use of non-linear least squares minimization. However, some spectra still appear noisy

even after smoothing, such that the parameters needed to be adjusted to match the experimental results. The fitting parameters can be found in the table below.

**ID**	g **(meV)**	ω_σ**(eV)**	γ_a**(meV)**	P_a**(meV)**	γ_σ **(neV)**	P_σ**(meV)**	γ_ϕ**(meV)**
**Spec#1**	50.0	1.875	100	27.66	15.0	0.03	6.68
**Spec#2**	50.0	1.91	100	1.72	15.0	1.81	26.26
**Spec#3**	50.0	1.94	100	0.48	15.0	0.48	55.70
**Spec#4**	50.0	1.955	100	12.03	15.0	0.02	41.70
**Spec#5**	50.0	1.970	100	22.15	15.0	2.51	35.46
**Spec#6**	50.0	1.995	100	0.21	15.0	0.01	45.84
**Spec#7**	50.0	2.040	100	14.60	15.0	1.72	30.24

Table S1 Fitting parameters for Figure 3A with quantum model.

Additionally, we present two other Qdots coupled with the same plasmonic slit cavity, as shown in Figure S13. In Figure S13A, the initial resonance of a single Qdot is very close (but still red-shifted) to the slit resonance (similar to Figure 1E), and a typical anticrossing feature is clearly visible. A similar anticrossing feature can also be seen in Figure S13B, where we intentionally selected a more red-detuned Qdot (initial uncoupled resonance is shown in Figure S14). However, as the Qdot resonance shifts towards the blue due to photooxidation over time, the Qdot undergoes complete photobleaching, preventing further observation of the asymptotic behaviour.

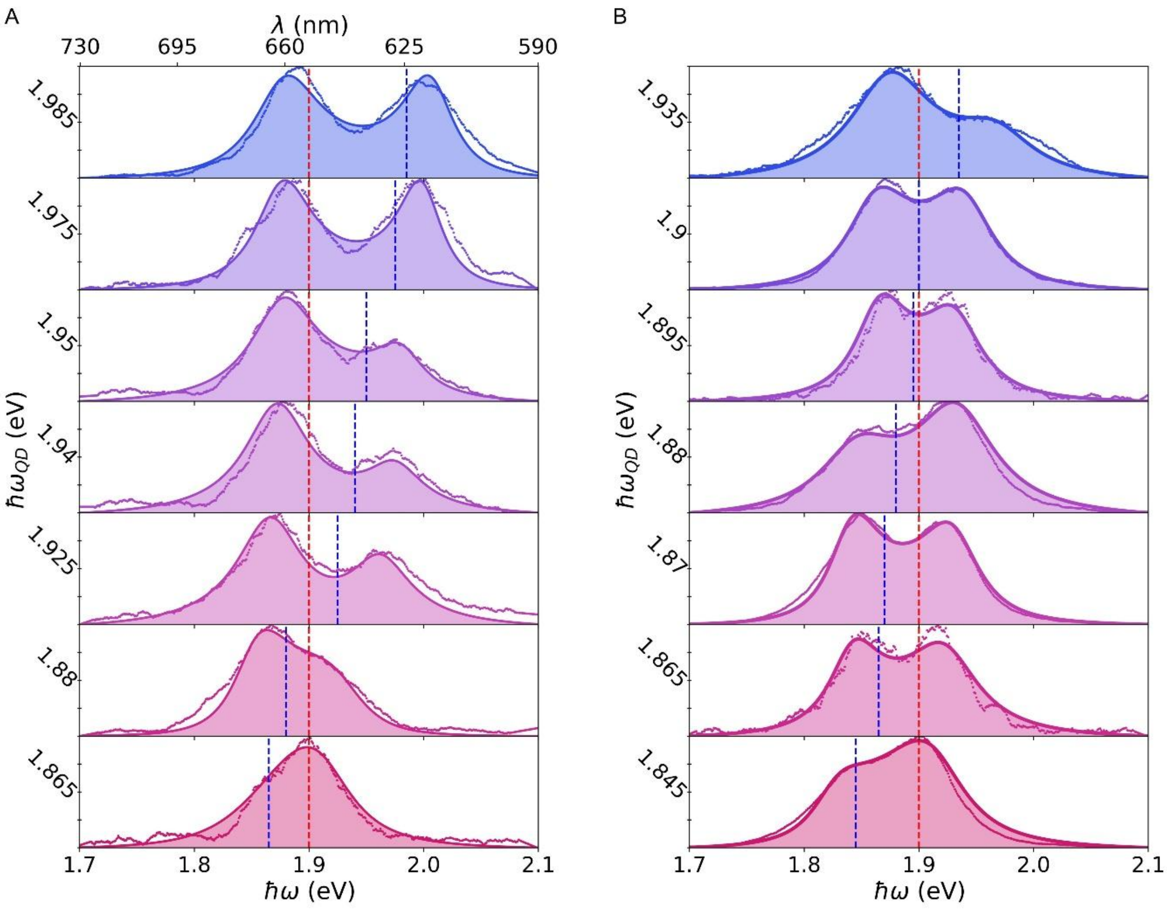

Figure S13 Another two datasets of typical anti-crossing behaviors in different single Qdots coupled with the same plasmonic slit cavity. The initial Qdot resonance in A) is similar as the one what we present in the main text and very close to slit resonance. In comparison, the Qdot resonance in B) is a little bit red detuned.

S7. Selection of the coupled spectra

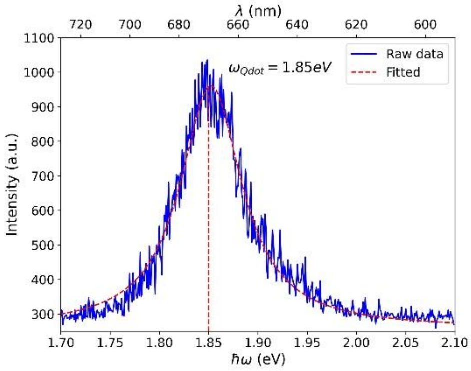

Figure S14 The initial resonance of the Qdot used in Figure S13B.

In the main text, the anticrossing spectra presented in Figure 3A were obtained from the sequential measurement based on their time sequence according to certain criteria (see below). A complete set of all coupling spectra in exact sequence recorded for a typical coupling experiment (raw data) is displayed in Figure S15, where the y-axis represents a strictly linear time progression. Each horizontal line corresponds to one measurement, with an integration time of 33 ms. The asymptotic behaviour of the high-energy polariton branch shows the linear blue shift with time which is induced by photon-oxidation. In a first processing step, the dark spectra, where the Qdot was in a "dim"-state or "off"-state and therefore showed no usable spectral features, were removed and the remaining spectra were smoothed and normalized. Importantly, in Figure S16A the y-axis no longer represents constant time intervals which leads to some distortion and a typical anticrossing behaviour may be difficult to discern. Overall, the spectra still follow the trend of increasing time for the positive y-direction. The two branches in the map illustrate how the coupling spectra evolve along the time sequence. The left branch starts from the lower energy and gradually converge to the PNR resonance as the initial resonance of Qdot is slightly red-detuned. The other branch shifts away to the higher energy due to the blue-detuned Qdot (caused by light-induced oxidation). Both features match the theoretical calculation quite well in Figure S16C. Note that the split spectra observed in the PL experiment can only be attributed to the coupled state and can't be due to Qdot clustering. This is because every selected single Qdot exhibits anti-bunching before the coupling experiments, which is still maintained after detaching the slit cavity and Qdots. Most importantly, direct emission of gold PL shaped by the PNR can be neglected as it is extremely week compared to Qdot emission.

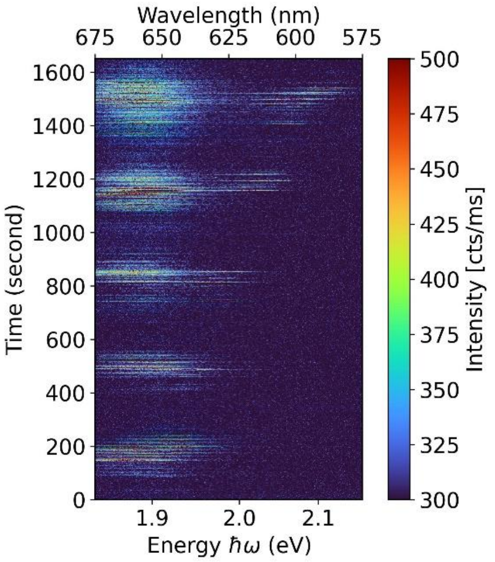

Figure S15 The raw data of anti-crossing experiment in Figure 3A including dark spectra.

The peak positions in the raw data in Figure S16A appear to undergo some fluctuations. This can be attributed to several factors. First, the primary reason is spectral diffusion of the quantum dot which is visible due to the time resolution of the experiment. Since the plasmonic slit cavity exhibits a broadband resonance (as shown in Figure S16D), there can be significant spectral overlap with the Qdot over a wide range of detunings, which causes spectral diffusion in the Qdot to reflect in both polariton lines. Moreover, the use of a scanning tip in combination with the plasmonic slit cavity may introduces small vibrations—though on the nanometer scale—which can significantly alter the relative position of the slit cavity and the Qdot, effectively impacting the coupling strength. Spectral diffusion of the bare quantum dot is also observed in the photo-oxidation experiment in Figure S16A. To obtain smoother data, the integration time can be extended to several seconds, as in other studies[21,22], and as demonstrated in Figure S16A. Here, we averaged the spectra over different rectangular areas in Figure S16A, yielding much smoother spectra with a clearly visible anticrossing feature. These averaged spectra can also be fitted using a quantum model. However, due to spectral diffusion and fluctuations in polariton intensity, the averaged curves are broadened, and in these fits, parameters such as cavity loss and coupling strength are generally overestimated and can vary.

To ensure unbiased data analysis, we selected additional four spectra from each of the seven marked areas in Figure S16A, chosen randomly and without bias, and fitted them using the quantum model, as illustrated in Figure S17. All four data sets indeed demonstrate the anti-crossing characteristic. The spectra in the same row of Figure S17 show similar polariton peaks, although the intensities may vary. This observation is further supported by theoretical calculations presented in Figure S18. In these calculations, all parameters are held constant except for the two pumping terms: cavity pumping and Qdot pumping. In Figure S18A-B, we observe that the two split peaks can vary in intensities when the cavity pumping terms are adjusted. A similar effect is evident when varying the atom pumping, as shown in Figure S18C-D.

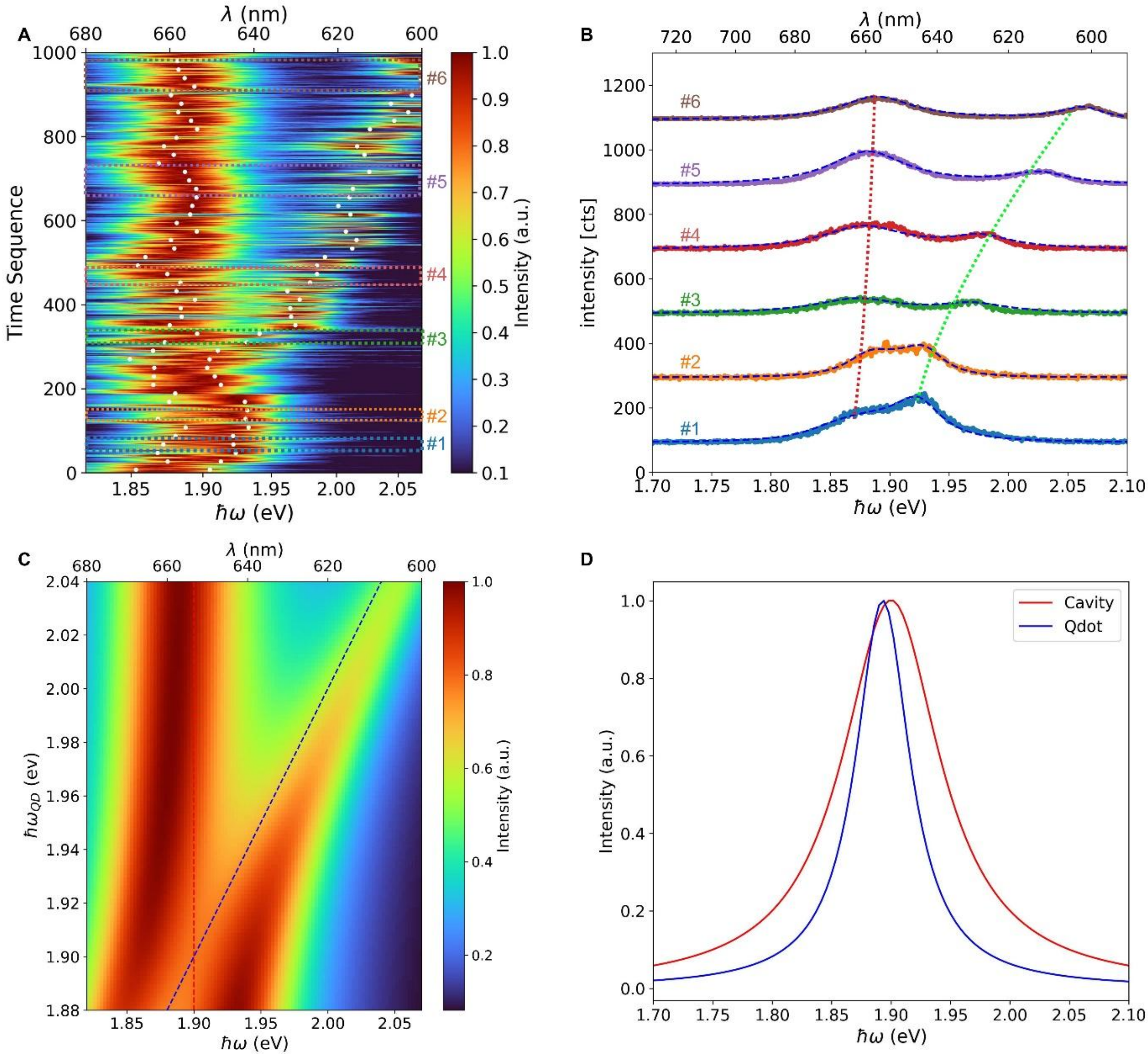

Figure S16 Coupled PL spectra arranged by time sequence. A) Time-sequenced anticrossing spectra dataset. The raw dateset is in Figure S15, and dark curves (indicating the Qdot “off” state) are removed. The y-axis in this case does not indicate equivalent time spacing, but still strictly follows the time trend. Spectra are divided by seven areas indicated by rectangular boxes. B) Averaged spectra from A within the dashed rectangles of the same color. The two branches labeled by dashed red and green curves are polaritons. C) The resized simulation map taken from Figure 3B. D) Spectral overlap between the Qdot and slit cavity.

In general, all the spectra can be fitted by the quantum model, and the corresponding Qdot resonance can be extracted. In Figure 3A we show only 7 spectra to demonstrate the typical anticrossing behavior with roughly equal additional energy shifts of Qdot resonance.

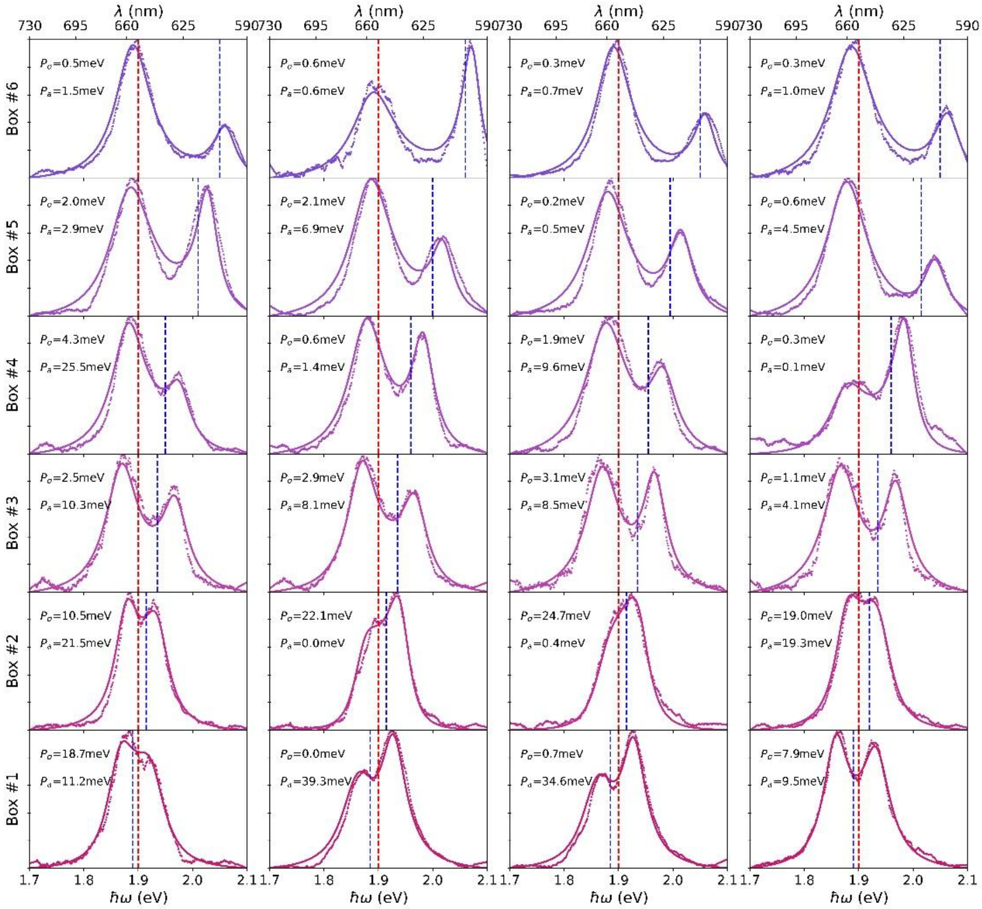

Figure S17 Another four groups of coupled spectra randomly selected form the rectangular boxes in Figure S16A. Spectra in the same row are picked from the same area.

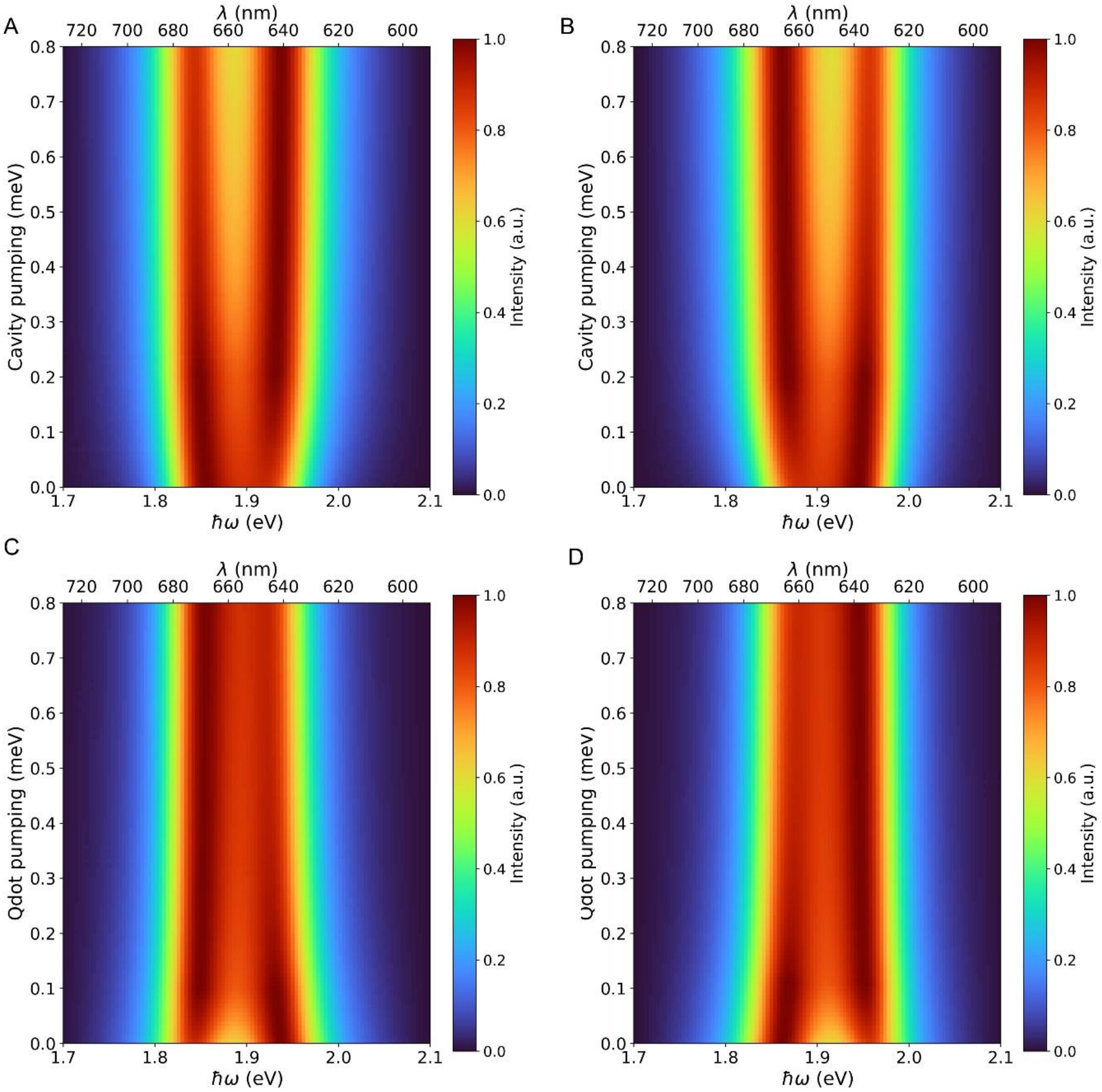

Figure S18 Coupled spectra simulated by quantum model. In all the calculations, cavity resonance ω_{σ}, coupling strength g, cavity decay γ_{a}, Qdot decay and dephasing (γ_{σ}, γ_{ϕ}) are fixed. A-B) Coupled spectra with different cavity pumping terms. Qdot resonance in A) is 1.88 eV and in B) is 1.92 eV. C-D) Coupled spectra with different Qdot pumping term. Qdot resonance in C) is 1.88 eV and in D) is 1.92 eV.

S8. Position dependent coupling experiments

The plasmonic slit cavity is integrated into the scanning probe tip, enabling us to spatially resolve the PL spectra and directly observe the transition from an uncoupled state to weak coupling, and eventually to strong coupling. As shown in Figure S19, we scanned a region containing a single Qdot using the tip-integrated plasmonic slit cavity and recorded the PL

spectra at each pixel. The selected spectra at representative positions are presented in Figure S19B.

At the bottom of Figure S19B, we show the PL spectrum of the bare Qdot, corresponding to the uncoupled regime. As the tip approaches the Qdot, the spectra exhibit broadening, an increase in intensity, and the emergence of a clear spectral splitting — signatures of enhanced coupling. After the tip passes over the Qdot, the splitting diminishes and the intensity decreases, consistent with reduced coupling. All spectra shown in Figure S19B are fitted using a quantum coupling model, from which we extract the corresponding position-dependent coupling strengths.

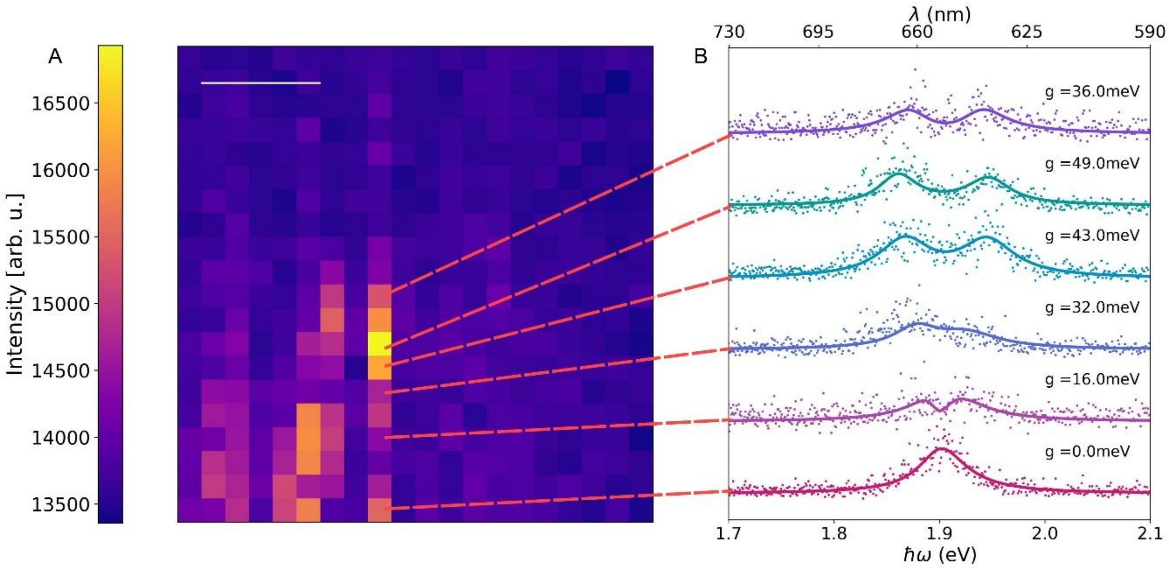

Figure S19 Position dependent Hyperspectral PL map. A), Hyperspectral PL map acquired using a scanning plasmonic slit cavity tip coupled to a single Qdot. Each pixel represents an individual PL spectrum, and the displayed intensities are obtained by integrating the spectral range from 630 nm to 645 nm. Scale bar: 50 nm. B) Corresponding PL spectra at selected labeled pixels, along with the extracted coupling coefficients obtained from fitting to a quantum model.

S10. Coupled-state photon statistic simulation

The coupled states, split into lower and upper polariton as indicated by the two branches in Figure S16A, are linear superpositions of excitons and photons. The fractions of excitons and photons in the polaritons are given by Hopfield coefficients $|X|^2$ and $|C|^2$ [23], which are defined as follows:

$$|X|^2 = \frac{1}{2}\left(1 + \frac{\Delta\omega}{\sqrt{\Delta\omega^2 + 4g^2}}\right)$$

$$|C|^2 = \frac{1}{2}\left(1 - \frac{\Delta\omega}{\sqrt{\Delta\omega^2 + 4g^2}}\right) \qquad \text{S9}$$

Here, $\Delta\omega$ is the energy detuning between the cavity resonance and the two-level system and g is the coupling efficiency. The decay rates of these two polaritons (γ_{LP}, γ_{UP}), are determined by the Hopfield coefficients, as they represent the fractions of excitons and photons. These decay rates are given by:

$$\gamma_{LP} = |X|^2\gamma_{QD} + |C|^2\gamma_a$$
$$\gamma_{UP} = |C|^2\gamma_{QD} + |X|^2\gamma_a \qquad \text{S10}$$

When the cavity and two level system are on resonance ($\Delta\omega = 0$), the decay rate simplifies to $(\gamma_{QD} + \gamma_a)/2$. In our experiment, the PNR exhibits significantly larger losses, which predominantly dictate the decay process of the polaritons. This indicates that polaritons tend to decay by emitting a photon, supporting our choice to use cavity emission for calculating the coupled spectra in the quantum model.

To solve the time dynamics of the polaritons, a simplified two-level rate equation model is used (See inset: Figure S20A):

$$\dot{p}_1(t) = \sigma_1 p_0(t) - P_1 p_1(t)$$
$$\dot{p}_0(t) = -\sigma_1 p_0(t) + P_1 p_1(t)$$
$$p_0(t) + p_1(t) = 1 \qquad \text{S11}$$

where $p_1(t)$ ($p_0(t)$) represents the probability of the polariton being present (absent). σ_1 and P_1 indicate the relaxation and pump rate of the polariton, respectively. The time-dependent photon emission rate $I(t)$ can be calculated by $\xi Q\sigma_1 p_1(t)$, where ξ describes the collection efficiency of the detection system and Q indicates the quantum yield. In simulations, we take $\sigma_1 = 1.9 \times 10^{13} Hz$, which means the polariton has an extremely fast decay time of approximately 52 fs.

The detection events can be artificially generated based on the time-dependent signals using Monte Carlo methods. Specifically, we first generate time-series photon signals with a certain time resolution, then sample the time-series signals with probability ρ, which creates the photon emission events. Since this involves single-photon emission, the detection events can only be assigned to one of the two collection channels. With this, the time-dependent detection events $I_{1,2}(t)$ can be generated and subsequently the second-order autocorrelation function is simulated by:

$$g^2(\tau) \propto \langle I_1(t) I_2(t+\tau)\rangle \qquad \text{S12}$$

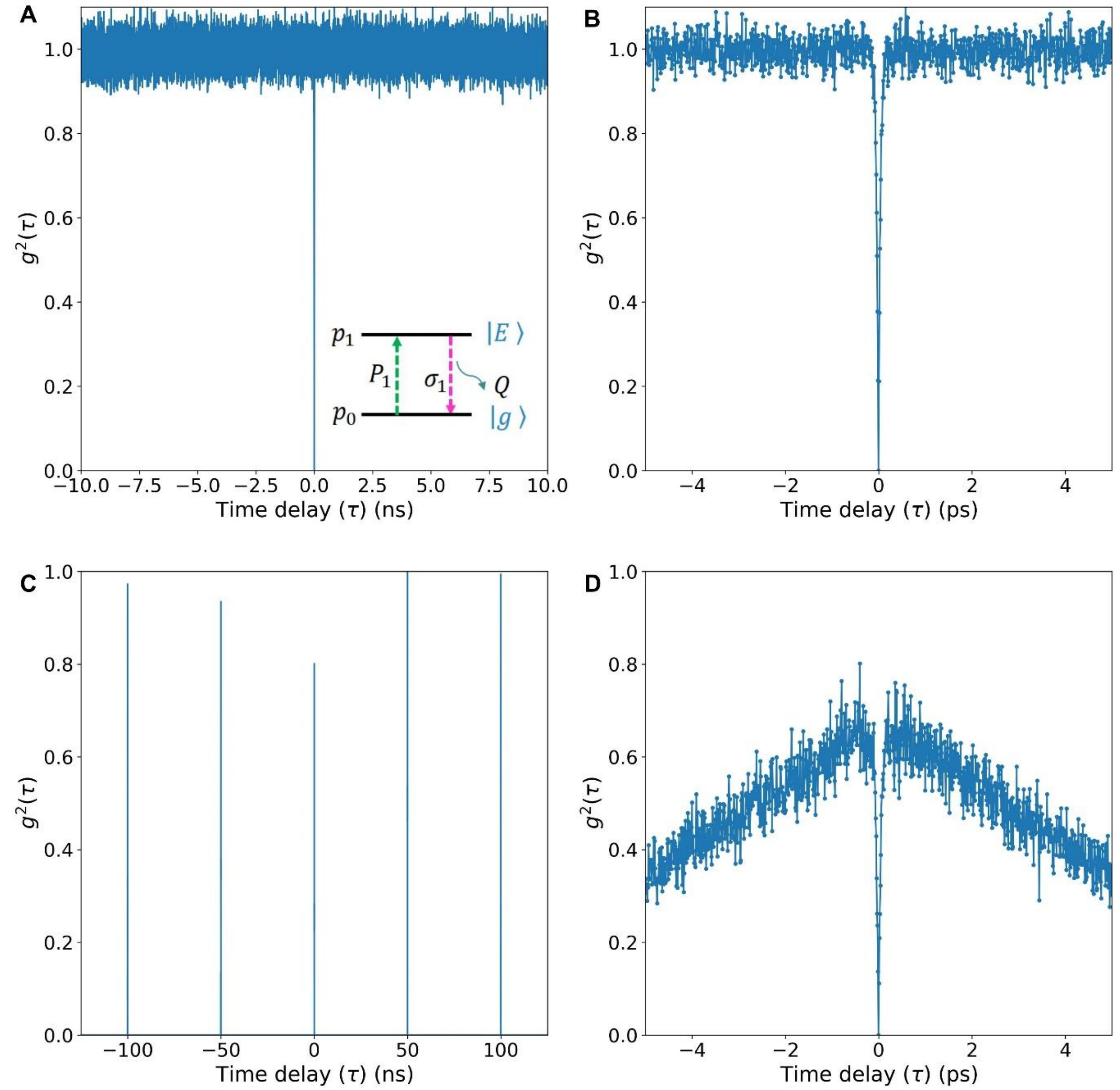

Figure 20 Photon statistic simulations based on Monte Carlo methods with different excitation methods. A-B) Normalized autocorrelation curve with CW excitation. Inset: the simplified two level system to describe the polariton generation and relaxation. B) is the zoom-in display in A), where we can resolve a dip indicates antibunching behaviour and very short lifetime. C-D) Normalized autocorrelation curve with pulsed excitation. From C), a typical bunching behaviour occurs. However, when zooming in the peak around zero time delay in D), a typical dip shows up.

Consider continuous-wave (CW) excitation first. As shown in Figure S20A-B, a dip appears at zero time delay with an extremely fast decay rate. This finite decay is only visible when zoomed in to the picosecond timescale because the lifetime of a polariton is roughly 52 fs. Unfortunately, resolving such a rapid decay in typical TCSPC experiments is practically

impossible due to the time-resolution limitations of photon-counting setups, which is on the order of hundreds of picoseconds. This limitation stems from the time jitter of photodetectors and electronic response times. Consequently, as depicted in Figure S20A-B, the dip at zero time delay below 0.5 is unresolvable in standard measurements.

An alternative approach is to utilize pulsed excitation. Here, one may employ a laser with a repetition rate of 20Mhz and a pulse width of 10ps. As shown in Figure S20C-D, a peak emerges at zero time delay if we bin the correlation events with a typical time resolution available in experiments (e.g., with few hundred picosecond accuracy). This is because when the polariton lifetime (52 fs) is significantly shorter than the pulse width 10ps, pulsed excitation closely resembles CW excitation. During a sufficiently long pulse duration, there is a significant chance for multiple polariton excitation and decay cycles to occur. Upon the excitation and subsequent decay of the first polariton via photon emission, the system immediately resets to the ground state. Thereafter, depending on the remaining pulse width, there is a certain probability of another polariton to be generated. In principle, anti-bunching still is maintained. When we zoom in the peak at the center shown in Figure S20D, the fast decay still is resolvable in simulations. However, such effects are impossible to see with typical, currently available setups.

Reference:

[1] J.-S. Huang, V. Callegari, P. Geisler, C. Brüning, J. Kern, J. C. Prangsma, X. Wu, T. Feichtner, J. Ziegler, P. Weinmann, M. Kamp, A. Forchel, P. Biagioni, U. Sennhauser, B. Hecht, *Nature Communications* **2010**, *1*, 150.
[2] X. Wu, R. Kullock, E. Krauss, B. Hecht, *Crystal Research and Technology* **2015**, *50*, 595.
[3] E. Krauss, R. Kullock, X. Wu, P. Geisler, N. Lundt, M. Kamp, B. Hecht, *Crystal Growth & Design* **2018**, *18*, 1297.
[4] H. Groß, J. M. Hamm, T. Tufarelli, O. Hess, B. Hecht, *Science Advances* **2018**, *4*, eaar4906.
[5] L. Brus, *Applied Physics A* **1991**, *53*, 465.
[6] M. Nirmal, B. O. Dabbousi, M. G. Bawendi, J. J. Macklin, J. K. Trautman, T. D. Harris, L. E. Brus, *Nature* **1996**, *383*, 802.
[7] W. G. J. H. M. van Sark, P. L. T. M. Frederix, A. A. Bol, H. C. Gerritsen, A. Meijerink, *ChemPhysChem* **2002**, *3*, 871.
[8] R. L. Olmon, B. Slovick, T. W. Johnson, D. Shelton, S.-H. Oh, G. D. Boreman, M. B. Raschke, *Physical Review B* **2012**, *86*, 235147.
[9] A. E. Schlather, N. Large, A. S. Urban, P. Nordlander, N. J. Halas, *Nano Lett.* **2013**, *13*, 3281.

[10] X. Xiong, Y. Lai, D. Clarke, N. Kongsuwan, Z. Dong, P. Bai, C. E. Png, L. Wu, O. Hess, *Advanced Optical Materials* **2022**, *10*, 2200557.
[11] C. Sauvan, J.-P. Hugonin, I. S. Maksymov, P. Lalanne, *Physical Review Letters* **2013**, *110*, 237401.
[12] R.-C. Ge, S. Hughes, *Opt. Lett.* **2014**, *39*, 4235.
[13] M. D. Leistikow, J. Johansen, A. J. Kettelarij, P. Lodahl, W. L. Vos, *Physical Review B* **2009**, *79*, 045301.
[14] A. Thränhardt, C. Ell, G. Khitrova, H. M. Gibbs, *Physical Review B* **2002**, *65*, 035327.
[15] B. W. Shore, P. L. Knight, *Journal of Modern Optics* **1993**, *40*, 1195.
[16] K. Słowik, R. Filter, J. Straubel, F. Lederer, C. Rockstuhl, *Physical Review B* **2013**, *88*, 195414.
[17] E. del Valle, F. P. Laussy, C. Tejedor, *Physical Review B* **2009**, *79*, 235326.
[18] E. del Valle Reboul, *PhD Thesis*, Universidad Autónoma de Madrid, **2009**.
[19] J. R. Johansson, P. D. Nation, F. Nori, *Computer Physics Communications* **2013**, *184*, 1234.
[20] A. Savitzky, M. J. Golay, *Analytical chemistry* **1964**, *36*, 1627.
[21] H. Leng, B. Szychowski, M.-C. Daniel, M. Pelton, *Nature Communications* **2018**, *9*, 4012.
[22] R. Chikkaraddy, B. de Nijs, F. Benz, S. J. Barrow, O. A. Scherman, E. Rosta, A. Demetriadou, P. Fox, O. Hess, J. J. Baumberg, *Nature* **2016**, *535*, 127.
[23] J. J. Hopfield, *Physical Review* **1958**, *112*, 1555.
